# In silico screening identifies a novel small molecule inhibitor that counteracts PARP inhibitor resistance in ovarian cancer

**DOI:** 10.1038/s41598-021-87325-5

**Published:** 2021-04-13

**Authors:** Z. Ping Lin, Nour N. Al Zouabi, Mark L. Xu, Nicole E. Bowen, Terence L. Wu, Ethan S. Lavi, Pamela H. Huang, Yong-Lian Zhu, Baek Kim, Elena S. Ratner

**Affiliations:** 1 Department of Obstetrics, Gynecology, and Reproductive Sciences, Yale University School of Medicine, New Haven, CT 06510 USA; 2 Department of Pediatrics, Yale University School of Medicine, New Haven, CT 06510 USA; 3 Yale West Campus Analytical Core, Yale University, West Haven, CT 06516 USA; 4 Department of Pediatrics, Emory University School of Medicine, Atlanta, GA 30322 USA; 5 Center for Drug Discovery, Children’s Healthcare of Atlanta, Atlanta, GA 30322 USA

**Keywords:** Cancer, Cancer therapy, Gynaecological cancer, Drug discovery, Virtual screening, Small molecules

## Abstract

Poly ADP-ribose polymerase (PARP) inhibitors are promising targeted therapy for epithelial ovarian cancer (EOC) with BRCA mutations or defective homologous recombination (HR) repair. However, reversion of BRCA mutation and restoration of HR repair in EOC lead to PARP inhibitor resistance and reduced clinical efficacy of PARP inhibitors. We have previously shown that triapine, a small molecule inhibitor of ribonucleotide reductase (RNR), impaired HR repair and sensitized HR repair-proficient EOC to PARP inhibitors. In this study, we performed in silico screening of small molecule libraries to identify novel compounds that bind to the triapine-binding pocket on the R2 subunit of RNR and inhibit RNR in EOC cells. Following experimental validation of selected top-ranking in silico hits for inhibition of dNTP and DNA synthesis, we identified, DB4, a putative RNR pocket-binding inhibitor markedly abrogated HR repair and sensitized BRCA-wild-type EOC cells to the PARP inhibitor olaparib. Furthermore, we demonstrated that the combination of DB4 and olaparib deterred the progression of BRCA-wild type EOC xenografts and significantly prolonged the survival time of tumor-bearing mice. Herein we report the discovery of a putative small molecule inhibitor of RNR and HR repair for combination with PARP inhibitors to treat PARP inhibitor-resistant and HR repair-proficient EOC.

## Introduction

Clinical approvals for poly(ADP-ribose) polymerase (PARP) inhibitors provide new treatment options for women with epithelial ovarian cancer (EOC). Olaparib is the first-in-class PARP inhibitor approved for the treatment of patients with advanced BRCA-mutated EOC who have been treated with three or more prior lines of chemotherapy. Thereafter, olaparib has been approved for the first-line maintenance treatment of patients with recurrent platinum-sensitive EOC regardless of BRCA status. Two more PARP inhibitors rucaparib and niraparib have been subsequently approved for similar indications in EOC.

PARP inhibitors demonstrate clinical efficacy by targeting BRCA mutations or defects in homologous recombination (HR) repair in breast and ovarian cancers^1,2^. PARP is a nuclear protein essential for the repair of DNA single strand breaks (SSBs)^3^. PARP binds to SSBs and catalyzes polymerization of ADP-ribose chains which signal and recruit other proteins to engage the repair process^4,5^. It has been suggested that inhibition of PARP leads to persistent SSBs^6,7^, which are converted into DNA double strand breaks (DSBs) by replication forks^8^. Thus, the resulting DSBs are necessarily repaired by HR^9,10^. It has been alternatively posited that HR repair is critical for replication restart or bypass of stalled replication forks in PARP-trapped SSB intermediates^11^. BRCA1 and BRCA2 proteins are critical components of the HR pathway. Therefore, cancer cells with BRCA1 and BRCA2 mutations or defects in the HR pathway are hypersensitive to the lethality of PARP inhibitors^12,13^.

However, the effectiveness of PARP inhibitors is limited to EOC with BRCA mutations (~ 15%)^14^ and about 50% of high-grade serous EOC that exhibits HR deficiency^15^. A significant portion of EOC remains resistant to PARP inhibitors. Furthermore, the reversion of mutated BRCA genes to restore HR repair function have been identified in both preclinical and clinical studies of EOC with acquired resistance to platinum and PARP inhibitors^16–18^. Given the growing use of PARP inhibitors, the cases of patients with PARP inhibitor resistant EOC is predictably on the rise.

To overcome the limitation of PARP inhibitors, we have been undertaking the discovery and development of small molecule inhibitors of HR repair aimed to the treatment of BRCA-wild type or HR-proficient EOC. Triapine is a small molecule inhibitor of ribonucleotide reductase (RNR) and 1000 times more potent than the clinically used RNR inhibitor hydroxyurea^19,20^. RNR is a heteromeric enzyme consisting of R2 and R1 subunits during the S phase of the cell cycle^21,22^. RNR catalyzes the rate-limiting step in the conversion of ribonucleoside diphosphates (NDPs) into corresponding deoxyribonucleoside diphosphates (dNDPs), the immediate precursors of deoxyribonucleoside triphosphates (dNTPs) essential for DNA replication and repair^23^. Triapine quenches the tyrosyl radical in the R2 subunit of RNR, thereby leading to enzymatic inactivation^24,25^. As a result, treatment with triapine promptly causes depletion of dNTPs and stalling of replicative DNA synthesis^26,27^.

Preclinical and clinical studies have demonstrated that triapine works effectively as chemo- and radio-sensitizer to augment the anticancer activity of DNA damaging agents and radiation^19,28–30^. With more than 80% clinical response rates in phase I/II studies^31–33^, Triapine is currently being studied in combination with cisplatin and radiation under a randomized phase III trial (NCT02466971) and a phase I trial (NCT02595879) in cervical and virginal cancers^32,34^. Triapine is well tolerated in patients and its side effects are generally manageable. The side effects of triapine include methemoglobinemia and dyspnea due to its strong iron-chelation property^35^.

Our mechanistic studies have demonstrated that triapine inhibits CDK activity, abrogates CtIP-mediated DSB end resection, suppresses HR repair, and sensitizes BRCA-wild type EOC to PARP inhibitors and platinum drugs in cell-based assays and tumor xenograft mouse models^29,30^. In keeping with our finding, independent studies also provide evidence that triapine blocks CtIP-dependent end resection of DBSs^36^, and inhibition of RNR by hydroxyurea suppresses HR repair^37^. We have also substantiated that depletion of the R2 subunit of RNR by siRNA suppresses the repair of endonuclease-induced DSBs by HR^29,30^. Furthermore, we have shown that the combination of triapine, olaparib, and cediranib effectively curbs subcutaneous growth and peritoneal progression of BRCA-wild type EOC xenografts and extends the survival time of mice^38^. Cediranib is a small molecule inhibitor of vascular endothelial growth factor (VEGF) receptor tyrosine kinases included in the combination to enhance the efficacy of olaparib and triapine.

In this present study, we sought to discover the next-generation RNR inhibitors to circumvent some pharmacodynamic and pharmacokinetic issues of triapine. Triapine is known to exhibit a short half-life in plasma (about 2 h) and hematological side effects linked to its iron-chelating property. Using the approach of in silico screening for hit enrichment and experimental validation of inhibitory potency, we have identified 2-[(4-{4-allyl-5-[(3-chlorobenzyl)thio]-4H-1,2,4-triazol-3-yl}-1-piperidinyl)methyl]-1,3-benzothiazole named DB4, a putative small molecule inhibitor of RNR that abrogates HR repair and sensitizes BRCA-wild type or HR repair-proficient EOC. This discovery provides a potential alternative to triapine and a new class of small molecule inhibitors for future drug design and development for ovarian cancer therapy.

## Results

### In silico screening of a compound library for docking the triapine-binding pocket on the R2 subunit of RNR

**Figure 1:**
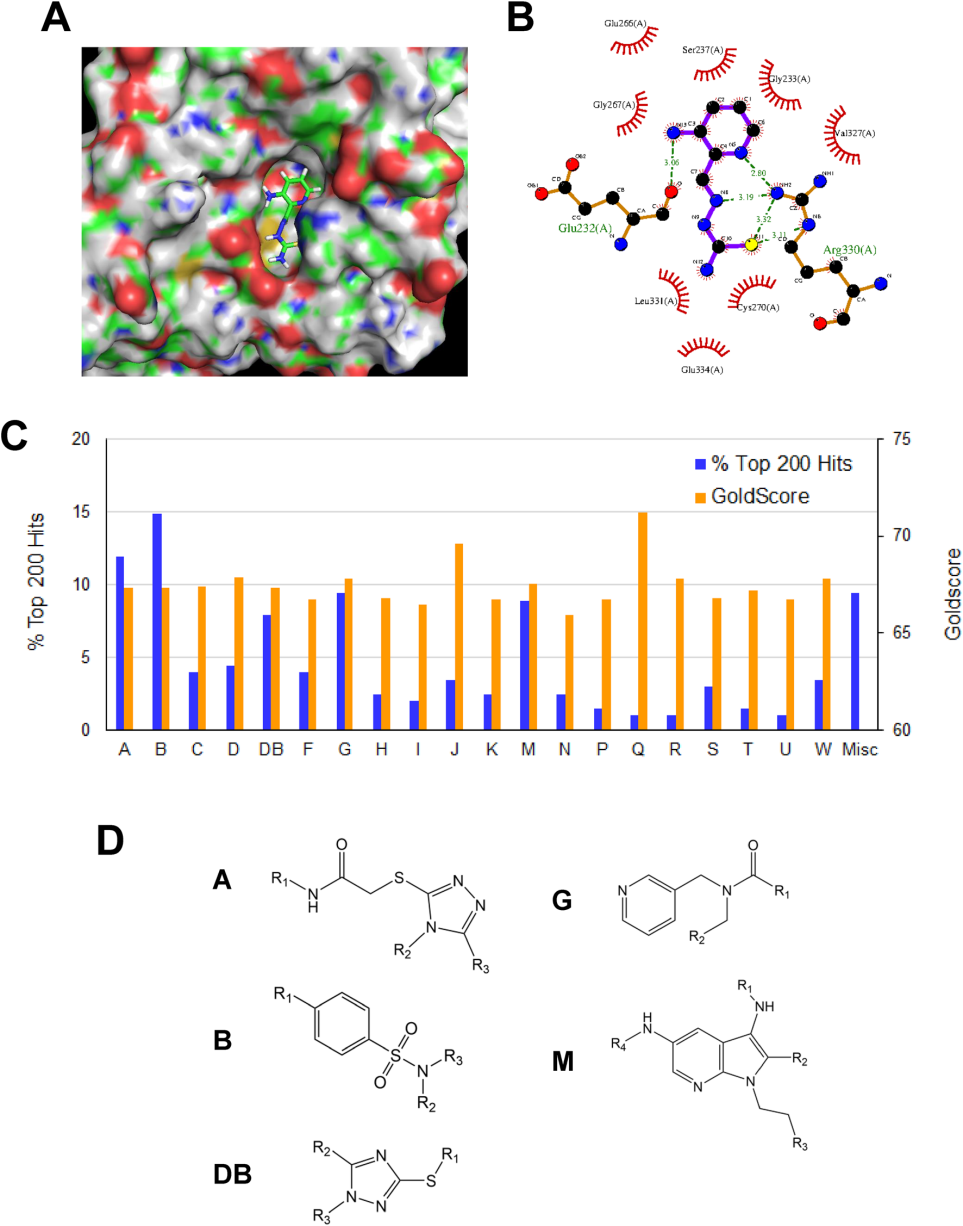
In silico screening and hit clustering for small molecule inhibitors of RNR. (**A**) Surface rendering for the triapine-binding pocket of the R2 subunit of RNR. A docking pose of triapine in the surface cavity of the putative triapine-binding pocket is modeled by the GOLD docking program and visualized using the PyMOL program. (**B**) The schematic diagram of molecular interactions between triapine and the triapine-binding pocket. The docking pose of triapine shown in A was run by the LigPlot+ program to generate the 2-D representation of 2 hydrogen bonds and 8 hydrophobic interactions with the binding pocket. (**C**) Hit clustering of top-ranked 200 compounds. These in silico hits were clustered to 20 groups. The percentage of hits of each group and the average of GOLDScore within each group are shown. (**D**) Structures of top 5 pharmacophore groups A, B, DB, G, and M. R1, R2, and R3 represent sides chain with varying structure in each group. Each group consists of 7–15% of top-ranked 200 compounds.

It has been postulated that triapine binds to a surface pocket of the R2 subunit of RNR^39,40^. This putative triapine-binding pocket positions in a close proximity of the di-ferric iron center and the tyrosyl-radical residue critical for the reduction activity of RNR^40^. It also lies in the interface between R2 and R1 subunits of the RNR complex^39^. Given its strong inhibitory activity toward RNR, triapine reportedly binds to this binding pocket of the R2 subunit to facilitate the inhibition of the reduction of ribonucleotide diphosphates (NDPs). We performed the docking of triapine into the triapine-binding pocket using the GOLD docking program (Cambridge Crystallographic Data Centre) and the rendering/modeling of molecular interactions using the PyMOL (Schrödinger, Inc.) and the LigPlot + (The European Bioinformatics Institute) programs. The results show that triapine potentially forms hydrogen bonds and several hydrophobic interactions with the triapine-binding pocket (Fig. 1A,B).

We conducted in silico screening of two subsets of the compound library (Chembridge) consisting of approximate 200,000 compounds using the GOLD program. The crystal structure of the R2 subunit of RNR was used for local docking of compounds into the triapine-binding pocket (Fig. 1A,B). The binding site was defined as the surface cavity centering Gly233 of the R2 subunit. Compounds were docked, scored and ranked according to their GOLDscores. We performed hit clustering of 200 top-ranking compounds based on structural and moiety similarities. Five distinct pharmacophores, each of which constitutes 7–15% of those 200 compounds were identified (Fig. 1C,D). Depending primarily on the commercial availability of these hits, total 25 compounds consisting of 3 to 9 compounds per pharmacophore group were arbitrarily chosen and acquired from Chembridge.

### Secondary screening of in silico hits by cell-based assays

**Figure 2:**
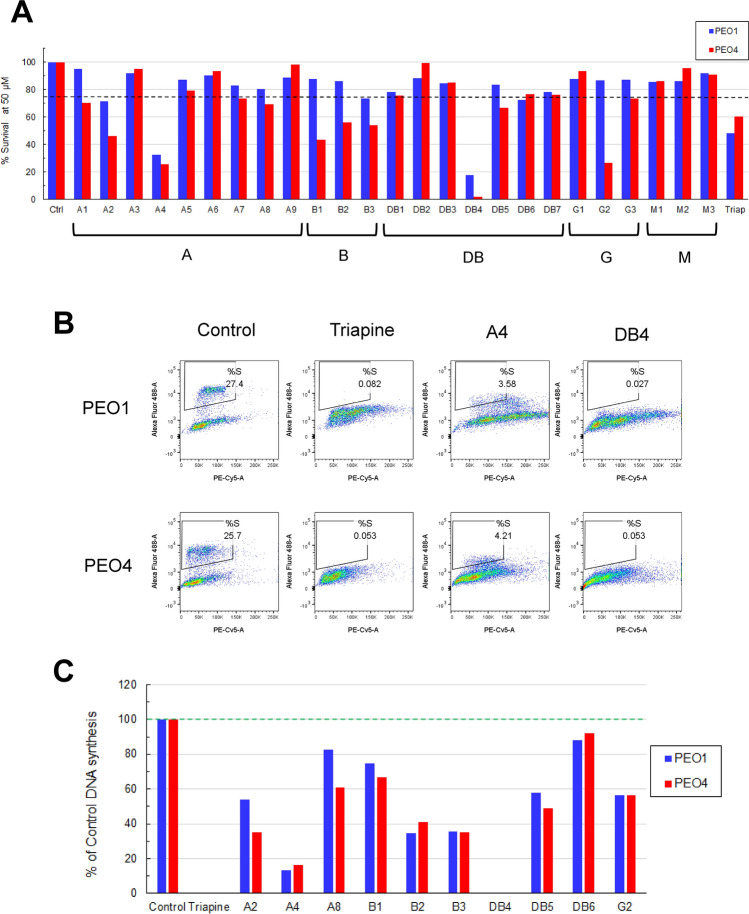

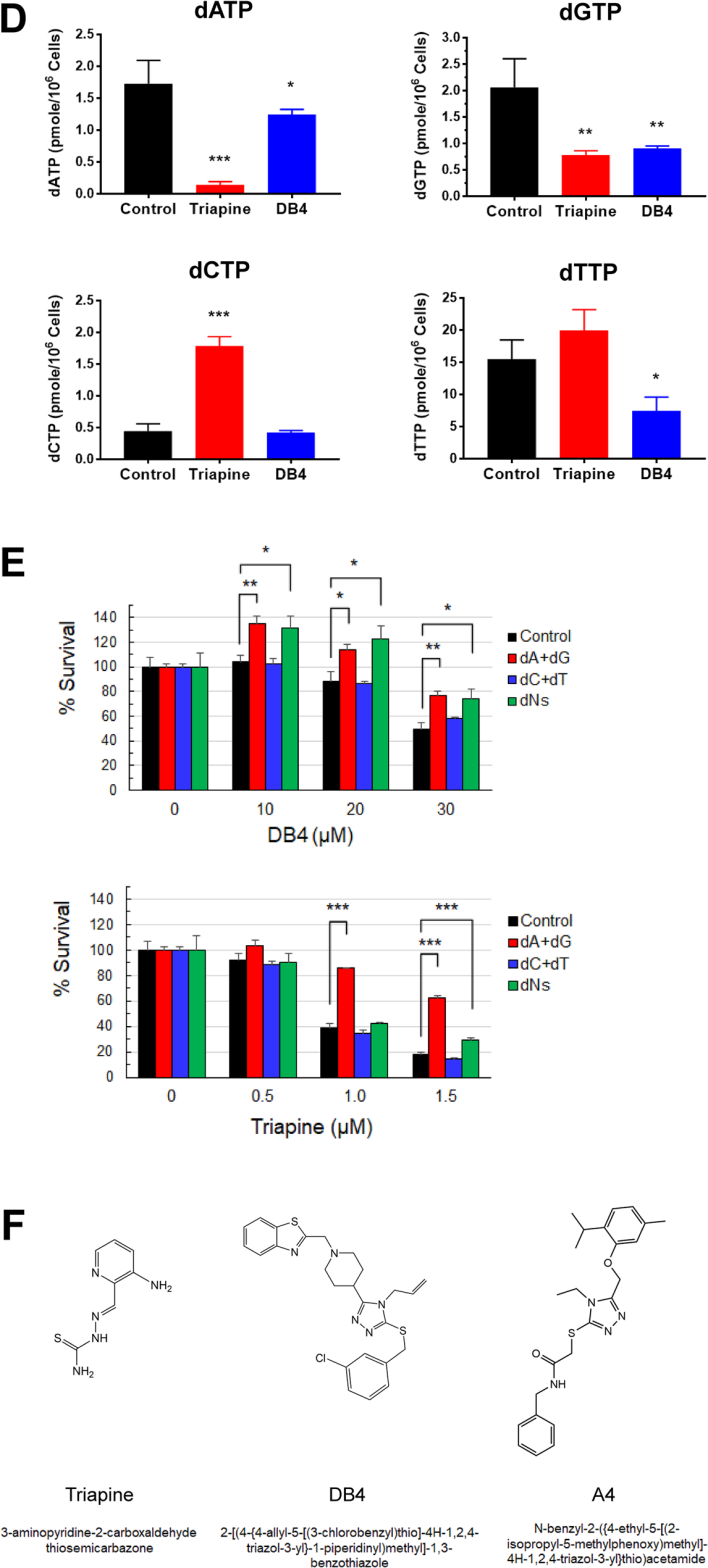
Follow-up assays of in silico hit compounds in PEO1 and PEO4 cells. (**A**) Cytotoxicity assays on selected 25 in silico hit compounds in PEO1 and PEO4 cells. Cells were plated for 24 h and then treated with 50 μM compounds for 72 h. For a positive control, cells were treated with 1 μM triapine for 72 h. The percentage of cell survival was determined by the MTS cytotoxicity assay. (**B**,**C**) DNA synthesis assays on 10 compounds that caused a 25% or more decrease in cell survival from the MTS assay. Cells were treated with 50 μM compounds or 1 μM triapine for 24 h. During the last hour, cells were pulse-labeled with EdU, stained with the Click-iT EdU Alexa Fluor 488 Assay Kit, counterstained with 7-AAD, and subsequently analyzed by flow cytometry. EdU-positive cells were gated to determine the percentage of the S phase cell population. The bivariate plots of Alexa Fluor 488 (EdU-positive) and PE-Cy5 (7-AAD-positive) are shown in (**B**). The percentages of DNA synthesis in cells treated with each of 10 compounds and triapine relative to the DMSO-treated control is calculated and shown in (**C**). (**D**) dNTP measurement on triapine and DB4 in PEO4 cells. Cells were treated with 1 μM triapine or 50 μM DB4 for 24 h. Cells were extracted with 65% methanol for determination of each of dNTPs expressed as pmol/10^6^ cells. Data are means ± SD (N = 3). *p < 0.05; **p < 0.01; ***p < 0.001, compared with the DMSO-treated control in each dNTP. (**E**) Reversal of RNR inhibition by supplementing deoxyribonucleosides (dNs). PEO1 cells were pre-treated with 1 mM dA plus 100 mM dG, 10 mM dC plus 10 mM dT, or all dNs for 1 h and then treated with various concentrations of DB4 or triapine for 72 h. The percentage of cell survival was determined by the MTS cytotoxicity assay. Data are means ± SD (N = 3). *p < 0.05; **p < 0.01; ***p < 0.001, compared with the control in each concentration of DB4 or triapine. (**F**) Structures and molecular compound names of triapine, DB4, and A4.

Next, we performed screening of 25 hit compounds for their ability to inhibit cell proliferation at 50 μM using MTS cytotoxicity assay (Fig. 2A). Judged by causing equal to more than 25% inhibition of either PEO1 or PEO4 cells, 10 compounds were advanced and evaluated for the inhibition of DNA synthesis. Using the flow cytometric EdU incorporation assay, we identified the most active compound DB4 that caused nearly complete inhibition (> 99%) of DNA synthesis in a manner similar to triapine in PEO1 and PEO4 cells (Fig. 2B,C).

Furthermore, we performed the dNTP measurement to evaluate the inhibitory effects of DB4 on RNR compared with triapine. DB4 caused significant decreases in the levels of dATP, dGTP, and dTTP. Consistent with our previous findings^21,26,41^, triapine produced a significant and pronounced decreases in the levels of dATP and dGTP while causing a marked increase in dCTP and somewhat elevated dTTP (Fig. 2D). The increases in dCTP and dTTP levels caused by triapine may be attributable to the dominance of the pyrimidine salvage pathway in mammalian cells^42^. To determine whether exogenous sources of dNTPs can bypass the inhibitory effects of DB4 and triapine on RNR, deoxyribonucleosides (dNs) were supplemented in the medium in the presence of DB4 or triapine. dNs can be transported into cells and subsequently converted to dNDPs and then dNTPs for DNA synthesis^43^. The survival of cells was determined by the MTS cytotoxicity assay as a readout. The result show that addition of dA plus dG, or all dNs, partially reversed the inhibitory effects of DB4 and triapine on cell survival (Fig. 2E). In contrast, addition of dC plus dT had no effects and somewhat counteracted dA plus dG on reversing the effects of triapine, suggesting that a delicately balance among individual dNTP levels is required for faithful DNA synthesis. Nevertheless, the findings are consistent with the results of dNTP measurements (Fig. 2D), in which DB4 and triapine caused a predominant decrease in dATP and dGTP levels. Because DB4 consistently led to suppression of cell proliferation, DNA replication, and dNTP synthesis indicative of RNR inhibition, we chose DB4 for the following studies. DB4 is structurally unrelated to triapine and has no apparent features of known iron chelating compounds (Fig. 2F). A4 is the second active compound that loosely share structural similarity with DB4.

### Structural activity relationship (SAR) between DB4 and the triapine-binding pocket

**Figure 3:**
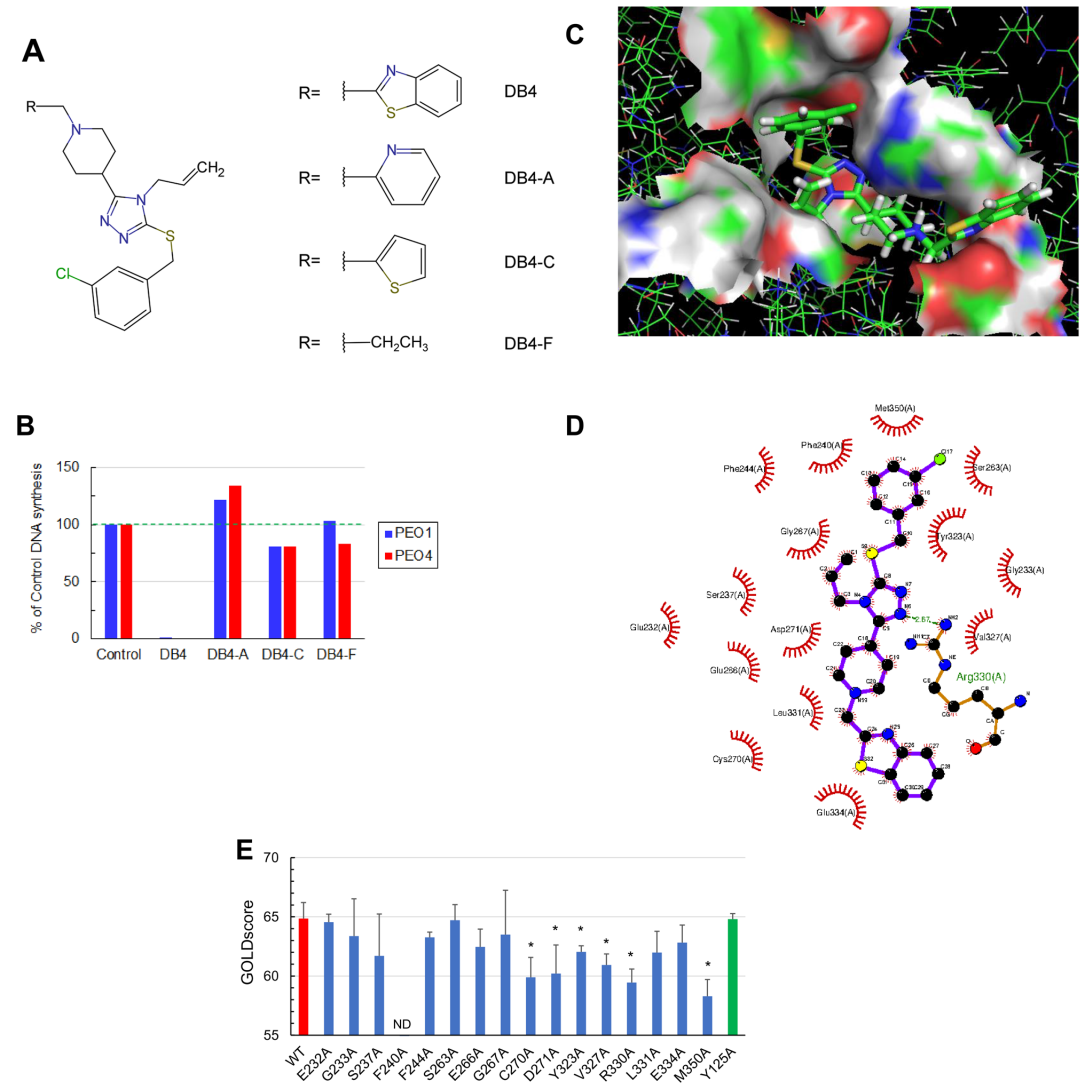
SAR of DB4 analogs and molecular modeling of DB4 bound to the R2 subunit of RNR. (**A**) Structures of DB4 and its analogs, DB4-A, DB4-C, and DB4-F. (**B**) Effects of DB4 analogs on DNA synthesis. PEO1 and PEO4 cells were treated with 50 μM DB4 or analogs and assayed for DNA synthesis inhibition as described in Fig. 2B. The bivariate plots of Alexa Fluor 488 (EdU-positive) and PE-Cy5 (7-AAD-positive) are shown in Fig S1. The percentage of S phase cells treated with DB4 or DB4 analogs relative to the DMSO-treated control is calculated and shown. (**C**) Surface rendering of the triapine-binding pocket and a putative docking pose of DB4. Only residues putatively interacting with DB4 are displayed in surface rendering. (**D**) The schematic diagram of molecular interactions between DB4 and the triapine-binding pocket. The docking pose of DB4 shown in C was run by the LigPlot+ program to generate the 2-D representation of 1 hydrogen bond and 15 hydrophobic interactions with the binding pocket. (**E**) Effects of in silico mutagenesis of the triapine-binding pocket on the docking scores of DB4. Sixteen key amino acid residues were mutated to alanine using the PyMOL program. DB4 was re-docked into each of mutated triapine-binding pockets using the GOLD program. The GOLDScores of top-ranking docking poses similar to the wild-type (WT) control were averaged. Data are means ± SD (N = 3). *p < 0.05 compared with the WT control. *ND* no similar docking poses detected.

We conducted similarity search for structural analogs of DB4 from Chembridge’s compound libraries. Three compounds of DB4 analogs were obtained for evaluating their ability to inhibit DNA synthesis compared with DB4 (Fig. 3A). These compounds retain the core structure of DB4 with its methyl-benzothiazole group being substituted with methyl-pyridine, methyl-thiophene, or propyl group, as DB4-A, DB4-C, or DB4-F, respectively. PEO1 and PEO4 cells were treated with these compounds for 24 h and DNA synthesis inhibition was determined by the flow cytometric EdU incorporation assay as described above. All DB4 analogs did not apparently inhibit DNA synthesis as opposed to DB4 in both cell lines (Fig. 3B and Fig S1).

To ensure that DB4 remained chemically stable, we performed the time course study by determining the potency of DB4 following a pre-incubation in the cell-free medium at 37 °C for up to 72 h. Cells were treated for 24 h with the medium containing pre-incubated DB4 and DNA synthesis was determined by the EdU incorporation assay. The results show that pre-incubated DB4 up to 72 h was more than 82% active to inhibit DNA synthesis in PEO4 cells (Fig S2A,B). Furthermore, the mass spectrometry analysis substantiates that the level of DB4 at 72 h remained similar to that of the 0 h control (Fig S2C).

To gain the insights into the molecular interactions between DB4 and the triapine-binding pocket, we performed re-docking of DB4 as described in Fig. 1 and modeled its potential binding pose. The results show that DB4 binds to the triapine-binding pocket through a hydrogen bond and an array of hydrophobic interactions (Fig. 3C,D and Table 1). Similar to triapine, DB4 forms a hydrogen bond with Arg330 of the binding pocket but lacks a hydrogen bond with Glu232. DB4 also shares all 8 hydrophobic interactions with triapine and possess additional 7 hydrophobic interactions with the binding pocket.

**Table 1:** Comparison of putative molecular interactions of the triapine-binding pocket with triapine and DB4.

Interaction type	Triapine	DB4
Hydrogen bond	Glu232	
	Arg330	Arg330
Hydrophobic Interaction		Glu232
	Gly233	Gly233
	Ser237	Ser237
		Phe240
		Phe244
		Ser263
	Glu266	Glu266
	Gly267	Gly267
	Cys270	Cys270
		Asp271
		Tyr323
	Val327	Val327
	Glu334	Glu334
	Leu331	Leu331
		Met350

To interrogate the importance of in silico interaction between DB4 and the triapine-binding pocket, we engineered mutations to disrupt key binding interactions implicated by the docking model. We performed in silico mutagenesis of 16 amino acid residues (Fig. 3C,D, Table 1) putatively interacting with DB4 in the triapine-binding pocket of the R2 subunit. Using the GOLD program, DB4 was re-docked into the binding pocket of each of mutated R2 subunits. The GOLDScore of three top-ranking docking poses (similar to that of Fig. 3C,D) were averaged and compared with that of the non-mutated control and a mutated negative control (Y125, a residue remote from the binding pocket). The results show that in silico mutations in 7 out of 16 amino acid residues led to a significant decrease in the GOLDScore (Fig. 3E), indicative of a reduced ability of DB4 to interact with the triapine-binding packet.

### Comparison of DB4 and triapine in modulating cell cycle distribution, CDK2 activity, and DSB end resection

**Figure 4:**
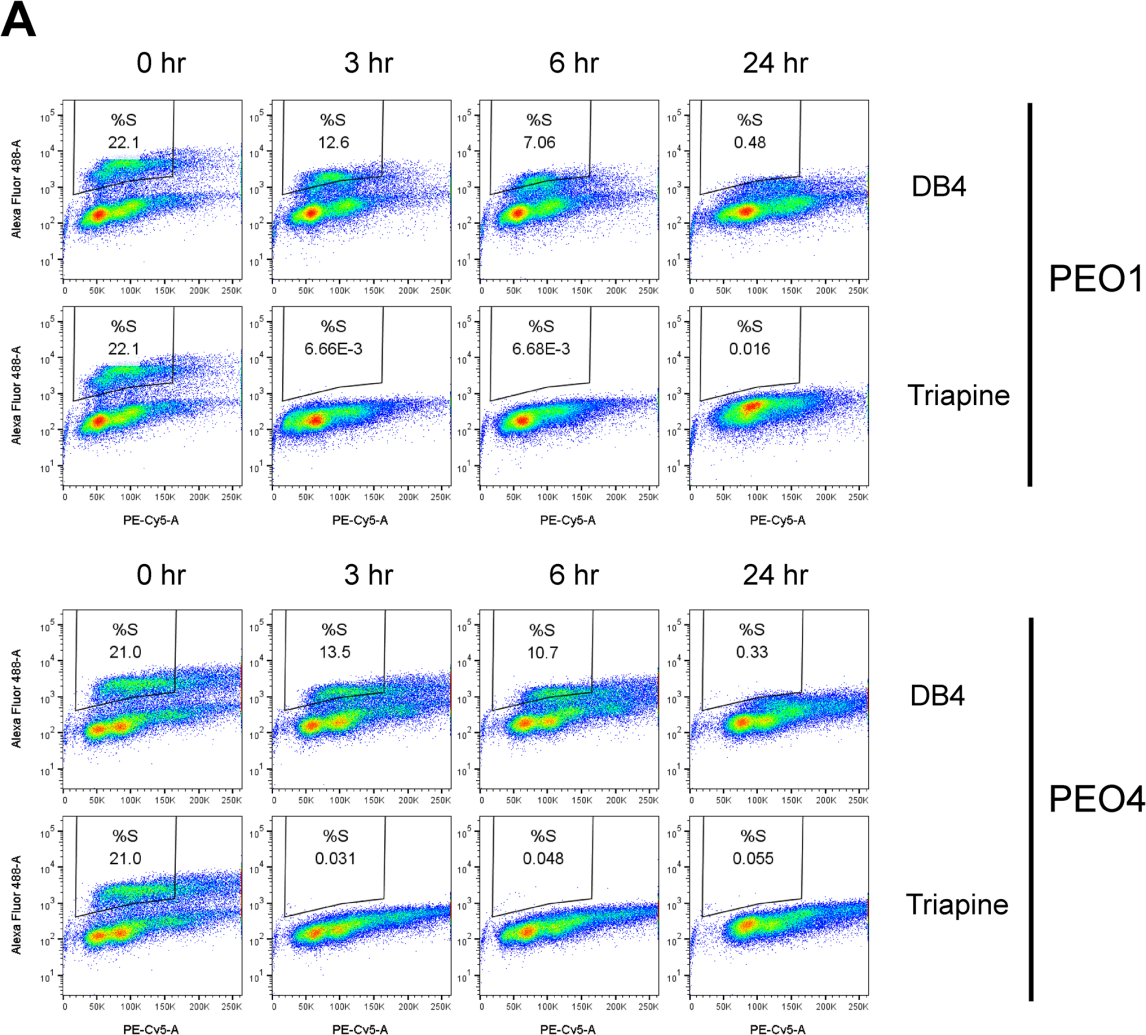

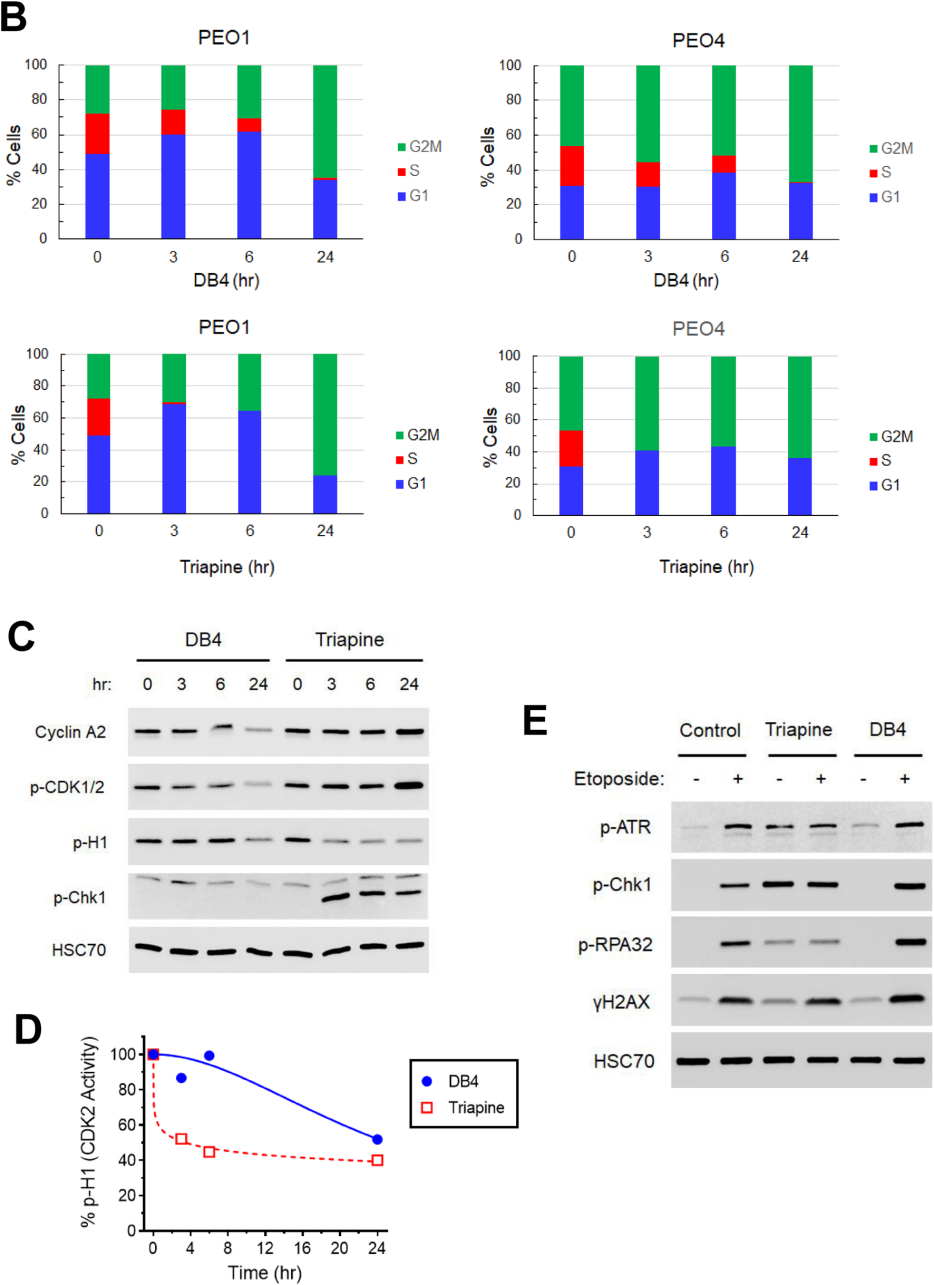
The time course of inhibition of DNA synthesis and CDK2 activity by DB4 or triapine. (**A**) Time-dependent DNA synthesis inhibition by DB4 or triapine. PEO1 and PEO4 cells were treated with 30 μM DB4 or 1 μM triapine for indicated times, pulsed with EdU for 1 h and subsequently assayed for DNA synthesis inhibition as described in Fig. 2B. The bivariate plots of Alexa Fluor 488 and PE-Cy5 were gated to determine %S phase cells. (**B**) Changes in cell cycle distribution caused by DB4 or triapine. The bivariate plots of Alexa Fluor 488 and PE-Cy5 shown in A were gated to determine % cells in G1, S, and G2/M phases. (**C**,**D**) Time-dependent inhibition of CDK2 activity by DB4 or triapine. PEO4 cells were treated with 30 μM DB4 or 1 μM triapine for various time periods and subjected to western blot analysis for the levels of cyclin A2, p-CDK1/2, p-H1, p-Chk1, and HSC70. The HSC70 protein level was used as a loading control. Cropped gel images of protein bands are presented. Full-length gel images are shown in Fig S5A. Cyclin A2, p-CDK1/2, and p-H1 bands were from re-probing the same blot. p-Chk1 and HSC70 bands were from re-probing the same duplicated blot. The CDK2 activity is expressed by the percentage of the p-H1 levels relative to that of the 0 h control, as shown in (**D**). (**E**) The effects of DB4 and triapine on DSB end resection. PEO4 cells were pre-treated with 30 μM DB4 or 1 μM triapine for 1 h and then treated with 5 μM etoposide for 4 h. Protein was analyzed by western blotting for p-Chk1, p-ATR, p-RPA32, γH2AX, and HSC70. Cropped gel images of protein bands are presented. Full-length gel images are shown in Fig S5B. All protein bands were from re-probing the same blot. DSB end resection is assessed by the level of p-RPA32. The level of γH2AX was used to confirm DSBs induced by etoposide.

We performed a time course study to comparatively assess how DB4 and triapine affected DNA synthesis and cell cycle in PEO1 and PEO4 cells. Both cell lines displayed a time-dependent inhibition of DNA synthesis by DB4 or triapine (Fig. 4A). Triapine caused a prompt reduction in EdU-positive cells, detected at 3 h and remained very low at 24 h. In contrast, DB4 led to a gradual decline in the EdU-positive cell population, sustaining a partial reduction at 3 and 6 h and only becoming very low at 24 h. Furthermore, we performed the analysis of cell cycle distribution shown in Fig. 4A. Both DB4 and triapine caused an expansion of the G2/M population presumably stemmed from a reduction in G1 and S phase populations in PEO1 cells (Fig. 4B). Similar phenomenon was also found in PEO4 cells except that the G1 population remained relatively unchanged (30–35%). The S phase population of PEO4 cells appeared to incorporate to the G2/M population by 24 h.

We next determined how DB4 inhibited CDK2 activity in comparison with triapine. In PEO4 cells, DB4 downregulated the levels of cyclin A2 and CDK2 phosphorylation most prominently at 24 h (Fig. 4C). In contrast, triapine increased the levels of cyclin A2 and CDK2 phosphorylation evidently at 24 h. Because CDK2 phosphorylation is inhibitory, the level of phosphorylated histone H1, a substrate of CDK2, was determined as a readout of CDK2 activity^29^ to reconcile this discrepancy. DB4 shared a similarity with triapine as evidenced by down-regulation of phosphorylated histone H1 at 24 h. Triapine reduced the level of phosphorylated histone H1 earlier starting at 3 h, which correlated with the onset of Chk1 phosphorylation (Fig. 4C,D). However, DB4 did not induced Chk1 phosphorylation. The kinetics of CDK2 activity somewhat resembled to the level of DNA synthesis caused by DB4 and triapine. Thus, DB4 slowly decreased in CDK2 activity by down-regulating the level of cyclin A2, whereas triapine promptly reduced CDK2 activity by increasing Chk1-mediated inhibitory phosphorylation of CDK2.

To investigate the effects of DB4 on DSB end resection, the level of RPA32 phosphorylation, indicative of CDK2-dependent DSB end resection, was determined. Etoposide induces DSBs, leading to a marked increase in RPA32 phosphorylation in PEO4 cells (Fig. 4E). Triapine induced a minor RPA32 phosphorylation but blocked phosphorylation of RPA32 induced by etoposide. In contrast, DB4 had no effects on basal RPA32 phosphorylation and yet augmented etoposide-induced RPA32 phosphorylation. Triapine induced ATR and Chk1 phosphorylation independently of etoposide treatment whereas DB4 only synergized with etoposide to induce Chk1 phosphorylation. DB4 appeared to weakly induce ATR phosphorylation. The synergy in Chk1 phosphorylation between DB4 and etoposide may result from enhanced RPA32 phosphorylation or DSB end resection. Collectively, these findings suggest that DB4 causes a gradual reduction in CDK2 activity allowing occurrence of DSB end resection and subsequent Chk1 activation. In contrast, triapine rapidly inactivates CDK2 primarily through Chk1 activation thereby blocking DSB end resection.

### DB4 abrogated HR repair in GFP reporter and nuclear foci assays

**Figure 5:**
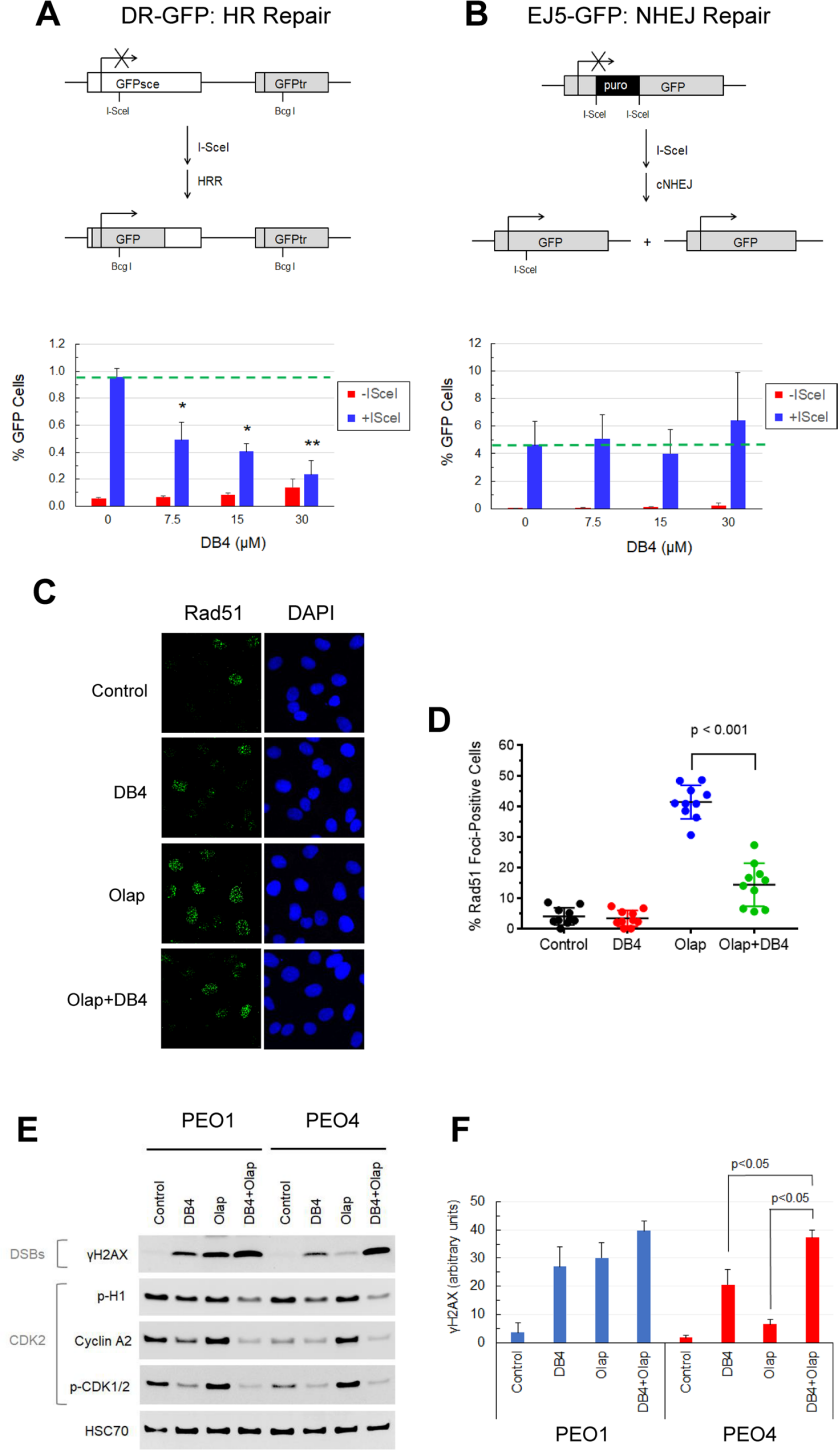
DB4 suppresses HR repair in BRCA-wild type SKOV3 cells. (**A**) Effects of DB4 on HR activity in SKOV3-DR-GFP cells. Cells were transfected with the ISceI-expressing plasmid and then treated various concentrations of DB4 for 48 h. Cells were harvested, stained with 7-AAD, and analyzed by flow cytometry for GFP-positive cells indicative of HR repair activity. (**B**) Effects of DB4 on NHEJ activity in SKOV3-EJ5-GFP cells. Cells were transfected with the ISceI-expressing plasmid and treated with various concentrations of DB4. Cells were harvested, stained with 7-AAD, and analyzed by flow cytometry for GFP-positive cells indicative of NHEJ activity. Data are means ± SD (N = 3). *p < 0.05; **p < 0.01, compared with the 0 µM control of ISceI-transected cells. (**C**) Confocal imaging of olaparib-induced nuclear Rad51 foci in SKOV3 cells treated DB4. Cells were treated with 30 μM DB4 for 1 h and then treated with 25 μM olaparib or 6 h. Cells were fixed, permeabilized, and stained with the anti-Rad51 antibody and the Alexa Fluor 488-conjugated secondary antibody. Nuclei were counterstained with DAPI. Immunofluorescence of Rad51 foci and nuclei were visualized by confocal microscopy. (**D**) DB4 significantly attenuates olaparib-induced Rad51 foci in SKOV3 cells. Cells were also scored for nuclei containing equal or more than 10 distinct Rad51 foci to determine the percentage of cells positive for Rad51 foci. Data are means ± SD (N = 10). p < 0.001, compared between indicated treatment groups. (**E**,**F**) Effects of DB4 on olaparib-induced γH2AX in PEO1 and PEO4 cells. Cells were pre-treated with 30 μM DB4 and then treated with 25 μM olaparib for 24 h. Protein was assessed by western blot analysis for the levels of γH2AX, p-H1, cyclin A2, p-CDK1/2, and HSC70. Cropped gel images of protein bands are presented. Full-length gel images are shown in Fig S6. p-H1, cyclin A2, p-CDK1/2, and HSC70 bands were from re-probing the same blot. The γH2AX band was from a duplicated blot. The band density of γH2AX indicative of DSBs was quantified by densitometry. Data are means ± SD (N = 3). p < 0.05, compared between indicated treatment groups.

We evaluated the ability of DB4 to inhibit endonuclease-induced DSB repair of reporter genes in SKOV3 cells. SKOV3 cells are proficient in HR and classical NHEJ (cNHEJ). SKOV3-DR-GFP cells were transfected with an I-SceI expression plasmid to induce a DSB in the DR-GFP reporter integrated in the genome for assaying HR repair activity, as described previously by our laboratory^29,30^. In addition, SKOV3-EJ5-GFP were transfected with an I-SceI expression plasmid to induce two DSBs in the EJ5-GFP reporter^44^ for assaying cNHEJ activity. SKOV3-EJ5-GFP cells have been established in our laboratory by stable transfection of the EJ5-GFP construct. Treatment of these SKOV3 cells with DB4 led to significant inhibition of HR repair induced by an I-SceI-induced DSB in a dose dependent manner (Fig. 5A). In contrast, DB4 appeared to enhance the cNHEJ activity induced by I-SceI-mediated DSBs but the increase was not statistically significant (Fig. 5B). The IC_50_ of DB4 for inhibiting HR repair was determined at 5.4 µM in SKOV3 cells. These results suggest that DB4 selectively inhibits HR-mediated DSB repair in SKOV3 cells.

To interrogate whether DB4 inhibited PARP inhibitor-induced DSB repair, nuclear Rad51 foci were determined in SKOV3 cells. Rad51 is a key component of the HR repair pathway. Treatment with DB4 had no effects on Rad51 foci, whereas treatment with olaparib induced a pronounced increase in Rad51 foci in SKOV3 cells. Importantly, DB4 significantly attenuated olaparib-induced Rad51 foci (Fig. 5C,D). These findings corroborate that DB4 inhibits HR repair in EOC cells.

Given that abrogation of HR repair hinders the recovery from DNA damage, we investigated whether DB4 enhanced the accumulation of olaparib-induced DSBs as measured by the level of γH2AX in BRCA-wild type EOC cells. The combination of DB4 and olaparib led to a pronounced increase in the level of γH2AX significantly greater than DB4 or olaparib alone in PEO4 cells (Fig. 5E,F). In contrast, due to a lack of HR repair, the combination of DB4 and olaparib produced a greater but not significant increase in γH2AX than DB4 or olaparib alone in PEO1 cells. As judged by the level of phosphorylated histone H1, DB4 caused a decrease in cyclin A2 and CDK2 activity corresponding to an increase in γH2AX, confirming HR impairment in HR-proficient PEO4 cells.

### DB4 enhanced DSBs and sensitized BRCA-wild type EOC to PARP inhibition

**Figure 6:**
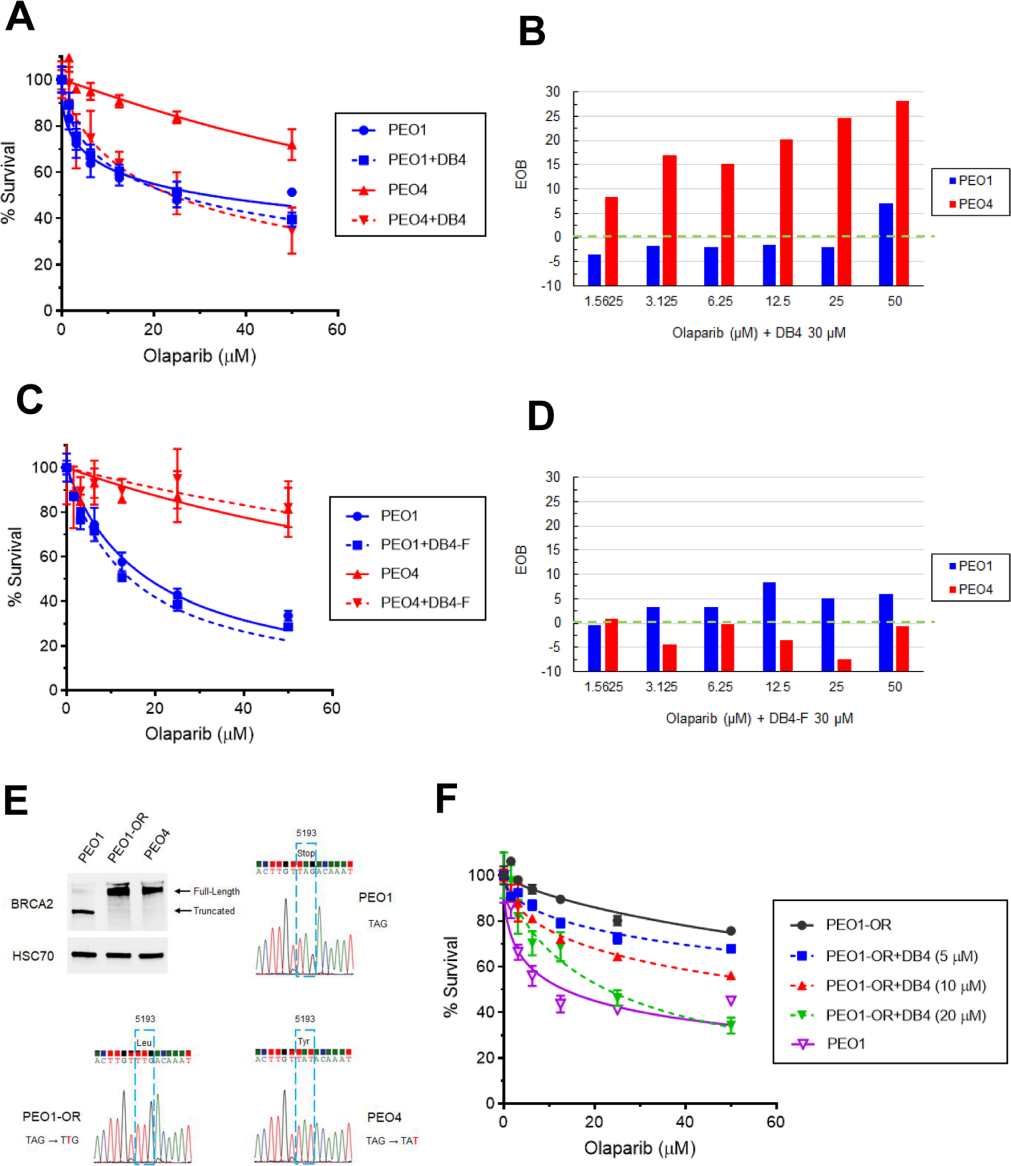

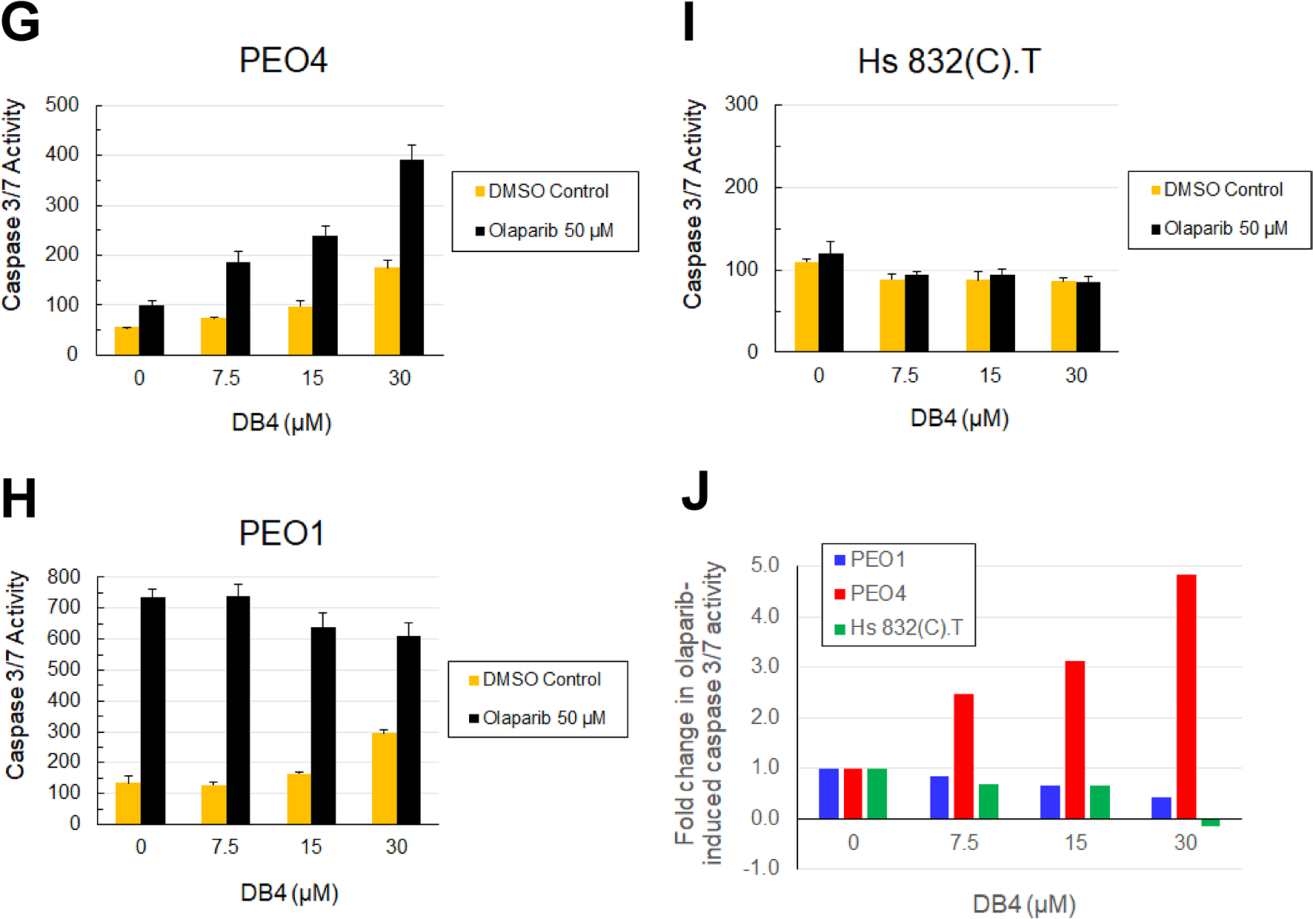
DB4 enhances olaparib-induced DSBs and sensitizes BRCA-wild EOC cells to olaparib. (**A**,**C**) Effects of DB4 or DB4F on the sensitivities of PEO1 and PEO4 cells to olaparib. Cells were pre-treated with 30 μM DB4 or DB4-F for 1 h and then treated with various concentrations of olaparib for 72 h. Cell survival was determined by the MTS cytotoxicity assay. Data are means ± SD (N = 3–4). (**B**,**D**) DB4 synergizes with olaparib to kill PEO4 cells. EOB calculation was used to quantify the synergy of the combination of DB4 or DB4-F and various concentrations of olaparib in PEO1 and PEO4 cells as shown in A and C. EOB > 0, synergism; EOB = 0, additivity; EOB < 0, antagonism. (**E**) Olaparib-resistant PEO1 (PEO1-OR) cells restore wild type BRCA2 expression by TAG to TTG reversion mutation at position 5193 nt. Olaparib-resistant PEO1 clones were examined by Sanger DNA sequencing and western blot analysis of BRCA2 expression compared with PEO1 and PEO4 cells. Cropped gel images of protein bands are presented. Full-length gel images are shown in Fig S7. BRCA2 and HSC70 bands were from re-probing the same blot. (**F**) Effects of various concentrations of DB4 on the sensitivity of PEO1-OR cells to olaparib. Cells were pre-treated with 0, 5, 10, or 20 μM DB4 for 1 h and then treated with various concentrations of olaparib for 72 h. Cell survival was determined by the MTS cytotoxicity assay. Data are means ± SD (N = 3–4). (**G**,**H**) DB4 and olaparib synergistically induces apoptosis in PEO4 but not in PEO1 cells. Cells were pre-treated with various concentrations of DB4 for 1 h and then treated with 50 µM olaparib for 24 h. Caspase 3/7 activity was expressed as RLU per µg/µL protein. Data are means ± SD (N = 3). (**I**) DB4 has no effects on olaparib-induced apoptosis in Hs 832(C).T cells. Cells were treated and caspase 3/7 activity was measured as described in (**G**) and (**H**). (**J**) The fold change of olaparib-induced caspase 3/7 activity with increasing DB4 concentrations is shown for PEO1, PEO4, and Hs 832(C).T cells.

We examined whether DB4 sensitized BRCA-wild type EOC cells to a PARP inhibitor. BRCA2-mutated PEO1 and BRCA2-wild type PEO4 cells were treated with DB4 at 30 µM in combination with various concentrations of olaparib and cell survival was determined. We found that DB4 effectively sensitized PEO4 cells to olaparib, while having minimal effects on PEO1 cells (Fig. 6A,B). Excess over Bliss (EOB) analysis for drug pair synergy also corroborated that the combination of DB4 and olaparib produced strong synergistic effects on PEO4 cells but had additive to slight antagonistic effects on PEO1 cells. In addition, we also tested the effects of a DB4 analog, DB4-F, on the sensitivities of PEO1 and PEO4 cells to olaparib. Because of a lack of inhibitory activity, DB4-F had no effects on the olaparib sensitivity of PEO1 cells and was unable to sensitize PEO4 cells to olaparib. (Fig. 6C,D).

We next varied the concentrations of DB4 to ascertain its effectiveness to cause sensitization to olaparib using a different BRCA-wild type EOC cell line. We established and used PEO1-OR cells, an olaparib-resistant PEO1 cell line derived from olaparib-sensitive PEO1 cells because of a reverted BRCA2 mutation and restored wild type BRCA2 expression (Fig. 6E). As expected, PEO1-OR cells were sensitized by DB4 to various concentrations of olaparib in a dose-dependent manner (Fig. 6F). PEO1-OR cells were partially sensitized by DB4 at 5 µM, and near-fully sensitized to olaparib at 20 µM as compared with PEO4 cells at 30 µM. Collectively, these results confirm that DB4 abrogates HR repair and therefore renders BRCA-wild type EOC cells hypersensitive to a PARP inhibitor.

Furthermore, the effects of DB4 on olaparib-induced apoptosis at 24 h were evaluated. Olaparib caused a minor increase in caspase 3/7 activity in PEO4 cells. DB4, starting at 7.5 µM, markedly augmented olaparib-induced caspase 3/7 activity in a dose dependent manner (Fig. 6G,J). In contrast, olaparib produced a pronounced increase in caspase 3/7 activity and DB4 failed to enhance olaparib-induced caspase 3/7 activity in PEO1 cells (Fig. 6H,J). Furthermore, the effects of DB4 on olaparib-induced apoptosis in non-malignant cells were investigated. Non-malignant cells may arrest the cell cycle at the G1 phase and curtail HR repair in response to olaparib treatment. As expected, DB4 did not enhance the apoptotic effects of olaparib on the benign ovarian cyst fibroblast cells Hs 832(C).T (Fig. 6I,J).

To further substantiate the effectiveness of DB4 and mitigate the issue of off-target effects with a higher concentration range of drugs used in the MTS cytotoxicity assay, we carried out clonogenic assays to determine the long-term survival of cells in response to treatments. The results showed that DB4 at 6 and 3 µM effectively reduced the clonogenic survival of BRCA-wild type SKOV3 EOC and MDA-MB231 breast cancer cells, respectively, in the presence of various lower concentrations of olaparib (Fig S3).

### The combination of DB4 and olaparib hindered the peritoneal and subcutaneous progression of olaparib-resistant EOC xenografts in mice

**Figure 7:**
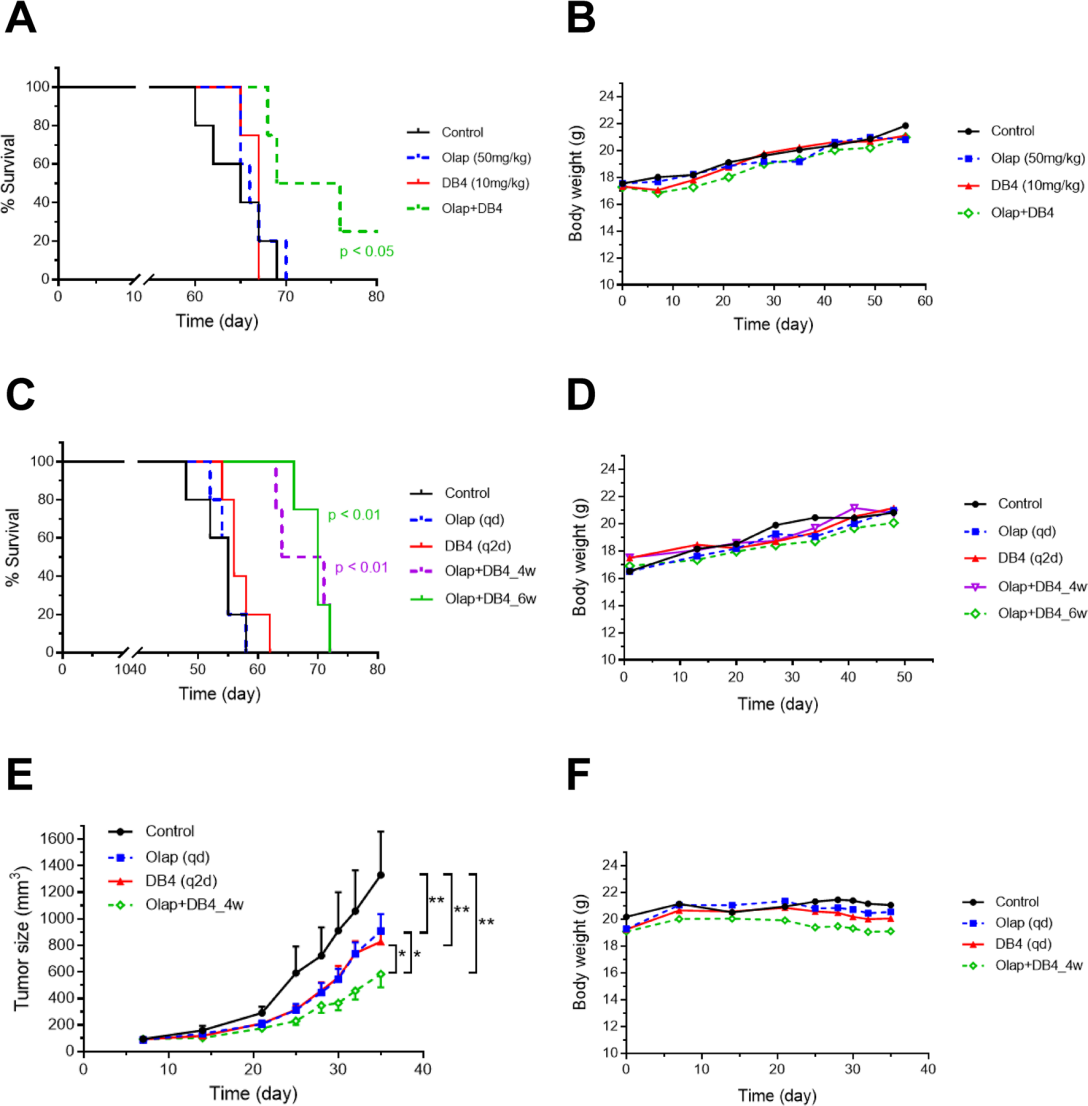
The combination of DB4 and olaparib suppresses the growth of BRCA-wild type EOC xenografts in mice. (**A**) The DB4-olaparib combination causes significant prolongation of the survival time of SCID-beige mice bearing with PEO4ip xenografts. SCID-Beige mice implanted ip with PEO4ip cells were treated daily (qd) with DB4 (10 mg/kg), olaparib (50 mg/kg), and the DB4-olaparib combination for 4 weeks. Kaplan Meier analysis was performed to determine the survival time of mice following treatments. Statistical significance was determined by the Mantel–Cox test comparing the control with each treatment group (n = 4–5). (**B**) The DB4-olaparib combination used in (**A**) exhibited no obvert toxicity to SCID-beige mice as determined by the body weight. (**C**) The modified DB4-olaparib combination furthers significant prolongation of the survival time of SCID-beige mice bearing with PEO4ip xenografts. DB4 given every two days (q2d) in the DB4-olaparib combination for 4 or 6 weeks (n = 4–5). (**D**) The DB4-olaparib combination used in (**C**) exhibited no obvert toxicity to SCID-beige mice as determined by the body weight. (**E**) The DB4-olaparib combination concertedly suppresses the sc growth of SKOV3 xenografts in mice. NCG mice implanted sc with SKOV3 cells were treated as described in (**C**) for 4 weeks during which the tumor size was measured. Statistical significance was determined by the Wilcoxon matched-pairs signed test comparing the control with each treatment group and comparing between treatment groups. Data are means ± SE (n = 4–5). *p < 0.05; **p < 0.01. (**F**) The DB4-olaparib combination used in (**E**) exhibited no obvert toxicity to NCG mice as determined by the body weight.

We have previously demonstrated that both PEO4ip and SKOV3 cells are BRCA-wild type and olaparib-resistant in cell-based assays and in vivo^29,30,38^. We first evaluated the efficacy of the combination of DB4 and olaparib to treat PEO4ip tumor xenografts, as determined by the median survival time, in a peritoneal mouse model^38^. Mice were implanted intraperitoneally (i.p.) with PEO4ip cells and then treated i.p. with olaparib, DB4, or both drugs in combination daily for 4 weeks. The survival endpoint (a 50% increase in abdominal circumference) for vehicle-treated control mice ranged from 55–65 days (8–9 weeks). Olaparib and DB4 alone had no effects on the survival time of mice (Fig. 7A and Table 2). In contrast, the combination of olaparib and DB4 moderately prolonged the survival time of mice (p < 0.05). Next, we repeated the experiment with modified treatment schedules of DB4 given every two day (q2d) for 4 and 6-week treatment periods. The combination of DB4 and olaparib produced significant and further extension of survival time of mice (p < 0.01) (Fig. 7C and Table 3). No apparent toxicity, as determined by changes in body weight of mice, was observed with either treatment schedules (Fig. 7B,D).

**Table 2:** Median survival time of PEO4ip xenograft-bearing SCID-Beige mice treated daily with olaparib, DB4, and both drugs in combination for 6 weeks.

Treatment	Median survival time (day)
Control	65
Olaparib (50 mg/kg, qd)	66
DB4 (10 mg/kg, qd)	67
Olaparib + DB4	73

**Table 3:** Median survival time of PEO4ip xenograft-bearing SCID-Beige mice treated with olaparib, DB4, and both drugs in combination for varied dose intervals and durations.

Treatment	Median survival time (day)
Control	55
Olaparib (50 mg/kg, qd)	55
DB4 (10 mg/kg, q2d)	56
Olaparib + DB4 (4 wks)	68
Olaparib + DB4 (6 wks)	70

We ascertain the efficacy of the combination of DB4 and olaparib to treat SKOV3 tumor xenografts, as determined by the tumor size, in a subcutaneous (s.c.) mouse model^30,38^. Mice were implanted s.c. with SKOV3 cells and then treated i.p. with olaparib, DB4, or both drugs in combination for 4 weeks in the same manner as Fig. 7C. Olaparib or DB4 alone moderately but significantly inhibited subcutaneous growth of SKOV3 xenografts (p < 0.01). Importantly, the combination of DB4 and olaparib resulted in concerted inhibition of tumor growth significantly greater than that caused by either olaparib or DB4 alone (p < 0.05) (Fig. 7E,F).

## Discussion

Using the in silico screening approach, we have identified DB4, a putative small molecule inhibitor of RNR that impairs HR repair for ovarian cancer therapy. This strategy for discovery of novel inhibitor compounds allows enrichment of hit compounds for subsequent experimental validation, providing a much greater advantage over traditional high throughput screening. Because only 25 out of 200,000 compounds were examined experimentally, our true hit rate was 4%, which is substantially higher than the overall hit rate of 0.0005%. In addition to experimental validation of identifying true hits, we also conducted analysis of physico-chemical properties of active compounds to enhance the likelihood of serving as a drug candidate. According to Lipinski’s rule of 5^45^, both DB4 and A4 exhibit appropriate “druglikeness” as defined as a molecular mass less than 500 Da, an octanol–water partition coefficient not exceeding 5, no more than 5 hydrogen bond donors, and no more than 10 hydrogen bond acceptors (Table S1). Topological polar surface areas are optimal (< 140), but aqueous solubilities are on the low side (< − 4)^46^ which leaves room for improvement of these hit compounds in future drug development.

In this study, we performed cytotoxicity and DNA synthesis inhibition assays as primary follow-up experimental validation of in silico hits. We took the advantage of the fact that inhibition of RNR by small molecule compounds, such as hydroxyurea and triapine, causes a pronounced reduction in cell proliferation and DNA synthesis. RNR mediates the rate-limiting step in the production of dNTPs necessary for DNA synthesis^21,26^. Our dNTP measurement and dNs supplementing assays further implicated that DB4 potentially inhibits RNR. However, we cannot completely rule out the possibility that DB4 acts independently of RNR inhibition. To address this issue, we are currently exploring in vitro binding assays and in silico molecular dynamics simulation to facilitate the follow-up validation processes for hit discovery.

DB4 may be one of the first-in-class of RNR inhibitors that does not involve quenching of tyrosyl-free radical for enzymatic inactivation. Notably, DB4 exhibits a distinct kinetics of DNA synthesis inhibition compared with triapine. We have previously shown that triapine rapidly depletes dNTPs, especially dATP and dGTP, and halts DNA synthesis within 1 h^21,26^. This is attributable to a prompt destruction of tyrosyl-free radical in the iron center of RNR^24^. In contrast, DB4 causes a gradual reduction in DNA synthesis over the period of 24 h. We speculate that triapine exhibits a greater potency and rate of inhibition by chemically inactivating RNR whereas DB4 reduces RNR activity by physically occupying the triapine-binding pocket of the R2 subunit. The function of the triapine-binding pocket remains largely unknown. It is hypothesized that the pocket serves as a channel involving the long-range proton-coupled electron transfer in RNR^47^. The free radical initiates from the iron center of the R2 subunit and propagates through the channel to the R1 subunit where the reduction of NDPs takes place. Moreover, the triapine-binding pocket positions in a close proximity to the interface between R2 and R1 subunits^47^. It is also possible that the binding of DB4 to the pocket disrupts the interaction of R2 and R1 subunits and hinders the formation of enzymatically active RNR. Nevertheless, future investigation into the enzyme kinetics of RNR is merited to characterize the differing mode of action between DB4 and triapine.

Our preliminary SAR study suggests that the benzothiazole group of DB4 is critical because its absence or other functional group substitutions abolish the inhibitory activity in DNA synthesis (Fig. 3B and Fig S1). Although DB4-A and DB4-C exhibit molecular interactions with the triapine binding pocket similar to DB4, the benzothiazole group of DB4 appears to endow additional hydrophobic interactions (Fig. 3D). In addition, the fused benzene ring of the benzothiazole group protruding above the triapine binding pocket may hinder the interaction between the R2 and R1 subunits. This structure is unique to DB4 compared with DB4-A and DB4-C (Fig S4). Nevertheless, this supposition requires future experimentation including site-directed mutagenesis of key amino acid residues in the triapine-binding pocket of R2 subunit in vitro, evaluation of more DB4 analogs with substitutions of other functional groups, and X-ray crystallography of the R2-DB4 complex to substantiate our proposed model for the molecular docking of DB4.

**Figure 8:**
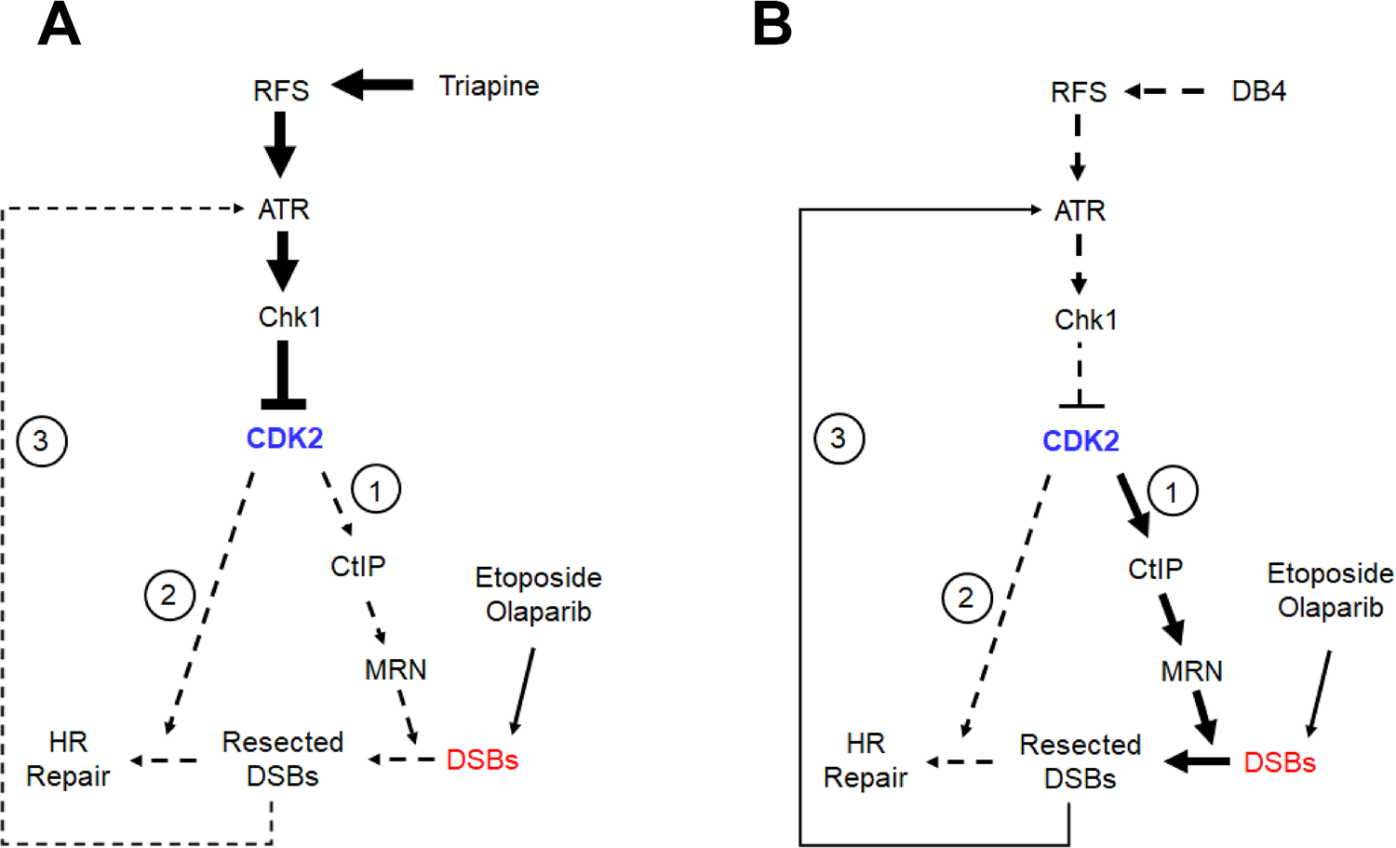
Proposed models of triapine (**A**) and DB4 (**B**) inhibiting CDK2 activity and HR repair. Triapine promptly and strongly inhibits CDK2 by ATR and Chk1, thereby blocking DSB end resection and HR repair predominately through routes 1 and 2 (in circle). DB4 is posited to gradually and weakly inhibit CDK2 and HR repair through route 2, allowing DSB end resection to occur (route 1). As a result, extensively resected DSB ends activate ATR and Chk1 to enforce the inhibition of CDK2 through route 3, leading to impairment of HR repair. RFS, replication fork stalling; Bold solid line, strong; Solid line, medium; Dash line, weak.

Furthermore, the difference in the rate of RNR inhibition caused by DB4 and triapine may provide an explanation for why DB4 does not abrogate DSB end resection in contrast to triapine. Because causing rapid depletion of dNTPs and stalling of DNA replication, triapine potently activates ATR and Chk1, thereby leading to prompt and complete inhibition of CDK2 and CtIP-mediated end resection of etoposide-induced DSBs^29^. In contrast, DB4 inhibits CDK2 activity but fails to block the end resection of DSBs caused by etoposide (Fig. 4E). We posit that DB4 causes dNTP depletion and CDK2 activity in a gradual fashion, thereby allowing DSB end resection to occur. Differing from triapine, the activation of Chk1 by combined DB4 and etoposide treatment may result from extensively resected DSBs. Thus, we propose the models of triapine and DB4-mediated inhibition of CDK2 and HR repair as depicted in Fig. 8. Given that CDK2 activity is required for activation of many key components of DNA damage response and HR repair pathways, such as phosphorylation of Nbs1 on Ser432^48^ and RNF4 on Thr26 and T112^49^, DB4 and triapine seem to be equally effective to inhibit HR repair regardless of their abilities to block DSB end resection. The inhibitory effects of DB4 on CDK2 substrates during S and G2 phases of the cell cycle is worthy of future investigation.

We demonstrate that DB4 not only sensitizes PEO4 cells but also PEO1-OR cells to the PARP inhibitor olaparib. PEO1 and PEO4 cells were derived from the same patient at first and second relapses, respectively, following platinum-based chemotherapy. Albeit being isogenic with altered BRCA2 and HR repair status, PEO4 cells may be clonally deviated from PEO1 cells because of tumor heterogeneity during disease progression in the patient. To mitigate this concern, we developed BRCA2-wild type PEO1-OR cells from BRCA2-mutated PEO1 cells in vitro to maintain the lineage of PEO1 background and minimize clonal variability and divergence. Our results substantiate that PEO4 and PEO1-OR cells can be effectively and equally sensitized to olaparib by DB4. DB4 indeed abrogates restored HR repair proficiency in these PARP inhibitor-resistant cells. Importantly, DB4 does not sensitize non-malignant ovarian fibroblast cells to olaparib, suggesting its specificity to EOC cells that heavily rely on HR repair for DNA damage and possibly its minimal toxicity to normal tissue.

Our in vivo studies show that DB4 synergizes with olaparib to hinder the progression of BRCA-wild type EOC xenografts and extend the survival time of tumor-bearing mice. It is noticeable that the combination regimen of DB4 given q2d is more efficacious than that of DB4 given qd to prolong the survival time of mice (Fig. 7B). Given the relatively slower kinetics of DB4, it is plausible that a reduced dosing frequency of DB4 is sufficient to achieve and maintain RNR/HR repair inhibition, with a less burden of RNR/HR inhibition-related toxicity to mice. Nevertheless, monitoring the body weight over the course of treatment strongly indicates this regimen is tolerable to mice even with severe immune deficiency. From clinical studies, triapine is known to have a short half-life in the plasma so a more frequent dosing schedule considers preferable. Furthermore, DB4 may be devoid of the adverse effects of triapine associated with iron-chelation including methemoglobinemia and dyspnea. Therefore, DB4 may provide some advantages of pharmacokinetics over triapine for treating patients.

In conclusion, we have identified DB4, a potential new class of putative small molecule inhibitor of RNR for HR repair impairment. Since the effective concentration of DB4 for sensitizing BRCA wild-type cancer cells to olaparib is at single-digit µM level, the principal structure of DB4 offers a promising starting point for design and development of more potent derivatives with improved pharmacokinetic properties. Furthermore, we demonstrate a proof-of-concept approach of using DB4 to leverage PARP inhibitors for the treatment of resistant or refractory EOC. Further characterization of the mode of action and improvement of the efficacy of DB4 will have meaningful impact on therapeutic innovation of EOC and provide a treatment option for patients who have PARP inhibitor-resistant EOC.

## Methods

### Cell lines and chemicals

SKOV3 ovarian cancer cell line (ATCC; Manassas, VA) was grown in McCoys 5A medium supplemented with 10% FBS and penicillin–streptomycin antibiotics. PEO1 and PEO4 ovarian cancer, Hs 832(C).T ovarian fibroblast, and MDA-MB231 breast cancer cell lines were grown in DMEM medium supplemented with 10% FBS and penicillin–streptomycin antibiotics. PEO1 and PEO4 cell lines were confirmed by short-tandem repeat (STR) analysis (Promega-ATCC). PEO1-OR cells were established by chronically exposing PEO1 cells to 2.5 µM olaparib for 4 weeks and propagating surviving olaparib-resistant PEO1 cells. Ascites-derived PEO4ip cell line was also authenticated by STR analysis (Yale DNA Analysis Facility on Science Hill). Hs 832(C).T and MDA-MB231 cells were purchased from ATCC (Manassas, VA). Olaparib was purchased from Selleck (Houston, TX). Triapine (3-aminopyridine-2-carboxaldehyde-thiosemicarbazone) was synthesized in our laboratory as previously described^30^. Screening compounds, DB4, and DB4 analogs were purchased from Chembridge (San Diego, CA).

### In silico screening, docking, and druglikeness analysis

In silico screening, docking, scoring, and ranking of hit compounds was performed using the GOLD docking program (Cambridge Crystallographic Data Centre; Cambridge, UK). The database files of the compound library were obtained from Chembridge (San Diego, CA). The PDB file of the R2 subunit of RNR (2UW2) was loaded and prepared (protonation and tautomeric states). The triapine-binding pocket^39,40^ using the Gly233 residue as the central point of the pocket was defined as the binding site for local docking. Two subsets of the compound database as the SDF file format were used for docking into the pocket. Each compound was docked for 10 times. GOLDScore was chosen for scoring the ligand fitness by protein–ligand hydrogen bond energy, protein–ligand and ligand internal van der Waals energy, and ligand torsion strain. Following the run, the GOLDScore of each screened compound was obtained and exported as an Excel file and ranked. Two hundred top-ranked hit compounds were manually clustered based on common pharmacophores and structural similarities. For visualization of docking poses and rendering of protein structures, the PyMOL program (Schrödinger, Inc., New York, NY) was used. The schematic diagrams of protein–ligand interactions were analyzed and generated from the docking poses files using the LigPlot+ program (The European Bioinformatics Institute). Physical and chemical properties of hit compounds was determined from the Chembridge database files using the DataWarrior program (http://www.openmolecules.org/).

### Cytotoxicity/viability assay

The assay was performed as described previously^21,26^. PEO1, PEO1-OR, and PEO4 cells were plated into 96 well plates for 24 h. Cells were then treated with drugs in triplicate or quadruplicated wells for 72 h. Thereafter, 20 µL of CellTiter 96 AQueous MTS Reagent (Promega; Madison, WI) was added to wells for additional 2 h incubation and the plates were immediately read by a colorimetric plate reader at wavelength of 490 nm. The absorbance was calculated to determine the percentage of cell survival relative to vehicle-treated controls.

### Clonogenic assay

The assay was conducted as described previously^29^. Cells were plated at various densities in 6-well plates in triplicate. After 24 h of incubation, cells were treated continuously with various concentrations of olaparib, DB4, or both drugs in combination. After 12–14 days, colonies were fixed/stained with crystal violet solution and counted to determine the percentage of survival using a GelDoc imaging system with QuantityOne software (Bio-Rad, Hercules, CA).

### DNA synthesis assay

During the final hour of drug treatment, cells were pulse-labeled with 10 μM EdU (5-ethynyl-2′-deoxyuridine). Cells were then collected, fixed, permeabilized for detection of S phase population using Click-iT EdU Alexa Fluor 488 Flow Cytometry Assay Kit (Thermo Fisher Scientific, Waltham, MA), followed by counterstaining of DNA with 7-aminoactinomycin D (7-AAD) (BD Biosciences, Franklin Lakes, NJ). Bivariate analysis of EdU incorporation and DNA content was performed by flow cytometry using LSRII flow cytometer (BD Biosciences) and FlowJo software (FlowJo LLC, Ashland, OR). The S phase cell population was gated to determine the percentage of cells undergoing DNA synthesis.

### dNTP measurement

The cellular levels of dNTP determination were carried out using the HIV-1 RT-based dNTP assay as described previously^50^. Approximate 2–3 × 10^6^ cells were treated with drugs for 24 h, trypsinized, counted, and washed with PBS twice. Cell pellets were extracted with 65% methanol at 95 °C for 3 min. The level of each of dNTP was determined and expressed as pmol/10^6^ cells.

### Liquid chromatography-multiple reaction monitoring-mass spectrometry (LC-MRM-MS) analysis

DB4 was dissolved in DMEM medium at 50 µM and incubated for 0, 24, 48, and 72 h at 37 °C and flash frozen after incubation. A 2 µL aliquot was injected and separated using an Agilent 1290 Infinity UPLC system with a reversed-phase column (Zorbax Eclipse Plus Rapid Resolution HD, 2.1 × 50 mm, 1.8 μm C18, Agilent) at room temperature. Mobile Phase A was 0.1% formic acid in water, mobile phase B was 0.1% formic acid in acetonitrile (LC–MS Optima Fisher). Gradient was 100% A for 1 min offline to desalt the sample, at 0.4 mL/min flow followed by a linear gradient to 98% B over 5 min at 0.4 mL/min. The qToF (Agilent 6550) was operated in positive scanning mode (50–1000 m/z) and the following source parameters: VCap: 3500 V, nozzle voltage: 2000 V, gas temp: 225 °C; drying gas 13 L/min; nebulizer: 20 psig; sheath gas temp 225 °C; sheath gas flow 12 L/min. Online mass calibration was performed using a second ionization source and a constant flow (5 μL/min) of reference solution (121.0509 and 922.0098 m/z). Compounds were identified based on the retention time of chemical standards and their accurate mass (tolerance 20 ppm).

### Western blot analysis

The methodology was described previously^29,30^. The anti-HSC70 antibody was purchased from Santa Cruz (Santa Cruz, CA). Anti-cyclin A2, anti-phospho-ATR (Thr-1981), anti-phospho-Chk1 (Ser-345), anti-phospho-CDK1/2 (Tyr-15), and anti-γ-H2AX (Ser-139) antibodies were from Cell Signaling (Danvers, MA). Anti-phospho-RPA32 (S4/S8) and anti-BRCA2 antibodies were from Bethyl Laboratories (Montgomery, TX). Anti-phospho-histone H1 antibody was from Millipore-Sigma (Burlington, MA). To avoid the cross-reaction of secondary antibodies with previously probed primary antibodies, blots were stripped and re-probed with next primary antibodies raised in a different species in an alternate fashion. All images were acquired and processed using the G: BOX gel documentation system and the GeneSnap software (Syngene, Frederick, MD). Brightness and contrast of images were applied equally across the entire gel/blot image and to controls. Images of protein bands were cropped and only brightness-adjusted using the PowerPoint software (Microsoft, Redmond, WA). Quantification of the protein band intensity was performed using the ImageJ software (NIH, Bethesda, MD).

### Caspase 3/7 assay

The assay was performed as described previously^38^. At the end of 24 h drug treatment, cells were lysed with the lysis buffer (PBS, 1% NP40, 0.1% SDS). 10 μL of lysate was incubated with Caspase-Glo 3/7 Assay reagent (Promega) at room temperature for 1 h and luminescence was subsequently measured with a luminometer (Turner Designs/Promega). Total protein concentration of cell lysates was determined as described above. Caspase 3/7 activity was expressed as luminescence units (RLU) per µg/µL protein for each sample.

### HR and NHEJ reporter assays

SKOV3-DR-GFP cells were established as described previously^29^. SKOV3-EJ5-GFP cells were established by stable transfection of SKOV3 cells with pimEJ5-GFP plasmid^44^ (Addgene, Watertown, MA). Cells were transiently transfected with the empty vector pRc/CMV/Neo (Thermo Fisher Scientific) or the I-SceI endonuclease expression vector pCBASceI (Addgene)^51^ using Lipofactamine 2000 (Thermo Fisher Scientific) according to the manufacturer’s protocol. Two hr after transfection, cells were treated with various concentrations of DB4 for 48 h. Cells were then trypsinized, stained with 7-AAD, and analyzed for the percentage of GFP-positive and 7-AAD-positive cells by flow cytometry using an LSR II flow cytometer (BD Biosciences) and FlowJo software (Tree Star; Ashland, OR). The ratio of %7-AAD-positive cells was used to normalize the percentage of GFP-positive cells.

### Immunofluorescent staining and confocal microscopy

SKOV3 cells were grown for 24 h prior to drug treatment. Cells were then fixed with 2% formaldehyde and permeabilized in cooled 100% methanol. The slides were blocked with 3% bovine serum albumin and stained with the rabbit primary anti-Rad51 antibody (Thermo Fisher Scientific) followed by incubation with the AlexaFluor 488- conjugated anti-rabbit secondary antibody (Thermo Fisher Scientific). Slides were then mounted with coverslips using the Prolong Gold Anti-Fade reagent (Thermo Fisher Scientific) containing DAPI for nuclei counterstaining. Immunofluorescence of Rad51 proteins and nuclei were acquired with a Leica SP5 confocal microscope (Wetzlar, Germany). Nuclei containing equal or more than 10 Rad51 foci were counted to determine % Rad51-positive cells.

### Excess over bliss (EOB)

Effects of two drugs in combination on cell survival were determined by EOB based on the principle of Bliss independence^52^. The value of EOB was calculated by the formula: EOB = (Ev − Cv) × 100, where Ev is the experimental value and Cv is the calculated value. Cv = Ea + Eb − Ea × Eb, where Ea is the fraction affected by drug a and Eb is the fraction affected by drug b. EOB > 0 indicates synergy, EOB = 0 indicates additivity, and EOB < 0 indicates antagonism.

### Statistical analysis

Statistical analysis for the effects of treatments in cell-based assays was performed by ordinary one-way ANOVA with Holm–Sidak's multiple comparisons test, using the Prism software (GraphPad, La Jolla, CA). All tests were two-tailed with an α level at 0.05.

### Tumor xenografts, drug treatments, and tumor growth evaluation

The Yale University Institutional Animal Care and Use Committee approved the protocol (IACUC# 2018-20038) for the in vivo animal studies in compliance with the US Public Health Policy on Humane Care and Use of Laboratory Animals. Yale University is registered as a research facility with the United States Department of Agriculture, License and Registration number 16-R-0001 The School of Medicine is fully accredited by the American Association for Accreditation of Laboratory Animal Care (AAALAC). An Animal Welfare Assurance (D16-00146) is on file with OLAW-NIH; Approval Period: May 1, 2019–May 31, 2023.

Five to 6 weeks old female SCID-Beige mice were purchased from Envigo (Indianapolis, IN). PEO4ip cells^38^ suspended in 100 µL serum-free medium were implanted i.p. (1 × 10^7^ cells per mouse). Six to 8 weeks old female NCG (NOD-Prkdc^em26Cd52^Il2r^gem26Cd22^/NjuCrl) mice were purchased from Charles River (Wilmington, MA). SKOV3 cells suspended in 100 μl serum-free medium were mixed with 50 μl Matrigel (BD Biosciences) and implanted s.c. in the right dorsal medial area (1 × 10^7^ cells per mouse). PEO4ip xenografts exhibited peritoneal progression as evidenced by ascitic development and abdominal distension. The abdominal circumference of PEO4ip-bearing mice reached a 50% increase about 50–70 days. SCID-Beige mice bearing PEO4ip xenografts exhibited 95% tumor take rate. NCG mice bearing SKOV3 xenografts displayed 100% tumor take rate.

Mice were randomly assigned to treatment groups (n = 4–5) and drug treatment was initiated 3–7 days after tumor implantation. The control group of mice received i.p. treatment with vehicle (2% DMSO) and treatment groups of mice received i.p. treatment for a period of 4 or 6 weeks as follows: Olaparib (50 mg/kg) once daily for 5 consecutive days per week; DB4 (10 mg/kg) once daily for 5 consecutive days (M, T, W, R, F) per week or once every 2 days (M, W, F) per week; The combined treatment with olaparib and DB4 at the dose and schedule described above.

The progression of PEO4ip tumor xenografts in mice was evaluated by measuring the size of the circumference at the lower one-third of the abdominal area with the mouse head positioned upward. A 50% increase in the abdominal circumference was defined as the endpoint of survival as described previously^38^. In contrast, the cohort mice without tumor implantation exhibited 15–17% increase in abdominal circumference at the time when vehicle treated PEO4ip-bearing mice reached a 50% increase in the abdominal circumference. Kaplan–Meier survival curve, median survival time, and statistical analysis was performed using the Prism software (GraphPad). Comparisons were made by the Mantel–Cox test comparing the control with each treatment group. The growth of SKOV3 tumor xenografts was evaluated by the size of s.c. tumor using a digital caliper. Tumor volumes were calculated using the formula: length × width^2^/2. Statistical difference in the tumor size was determined by the Wilcoxon matched-pairs signed test comparing the control with each treatment group and comparing between treatment groups. Body weights of mice were measured on every treatment day before administration and on the same schedule as tumor measurements during the treatment period.

## Supplementary Information

Supplementary Information.
