# Supplementary material for: In silico screening identifies a novel small molecule inhibitor that counteracts PARP inhibitor resistance in ovarian cancer
Source: Sci Rep. 2021 Apr 13;11:8042. doi: 10.1038/s41598-021-87325-5 (PMC8044145; doi:10.1038/s41598-021-87325-5)
Supplement: Supplementary file 1 — Supplementary Information. [file 41598_2021_87325_MOESM1_ESM.pdf]

SUPPLEMENTARY INFORMATION

In Silico Screening Identifies a Novel Small Molecule Inhibitor That Counteracts PARP inhibitor Resistance in Ovarian Cancer

Z. Ping Lin, Nour N. Al Zouabi, Mark L. Xu, Nicole E. Bowen, Terrence L. Wu, Ethan S. Lavi, Pamela H. Huang, Yong-Lian Zhu, Baek Kim, and Elena S. Ratner

Table S1. Predicted physical and chemical properties of A4 and DB4 analogs for evaluation of druglikeness. MW, molecular weight; cLogP, octanol/water partition coefficient; LogSW, intrinsic water solubility; RB, rotatable bond; tPSA, topological polar surface area; hDON, hydrogen bond donor; hACC, hydrogen bond acceptor.

	MW	cLogP	LogSW	RB	tPSA	hDON	hACC
A4	438.6	3.78	-7.00	8	69.0	1	5
DB4	496.1	4.74	-7.49	8	46.8	0	5

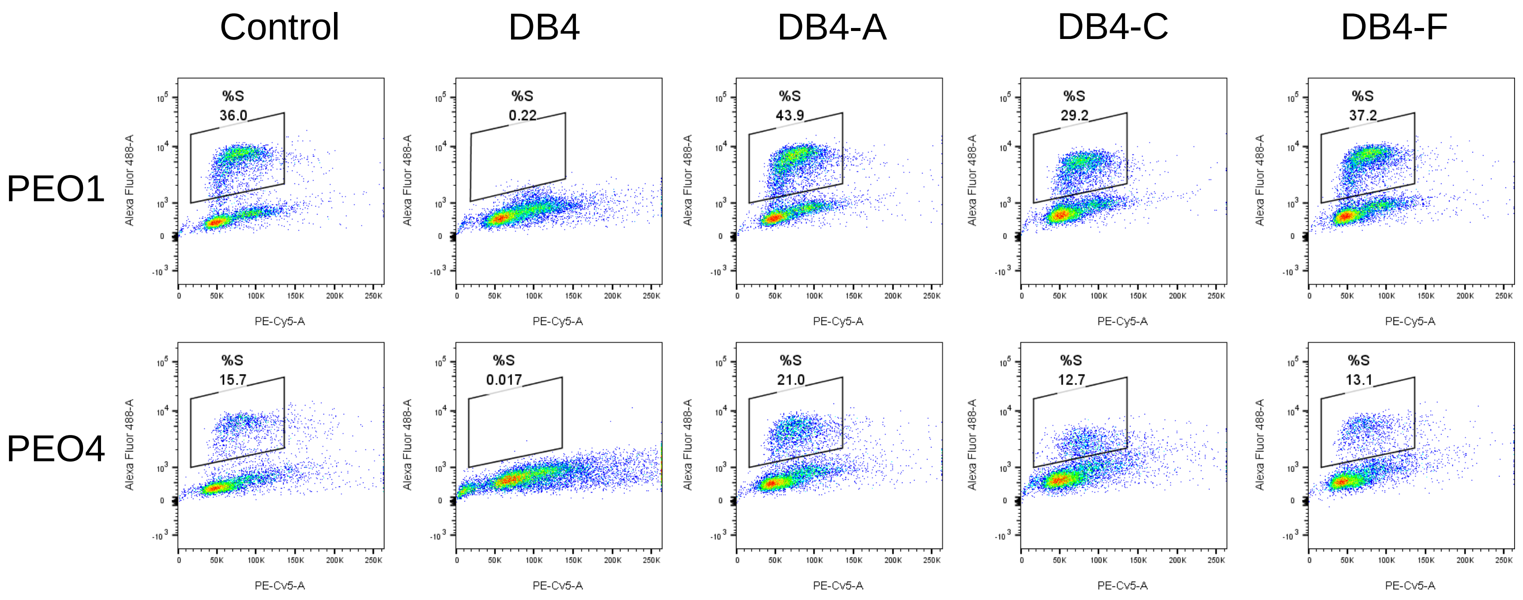

Fig S1. Effects of DB4 and its analogs on DNA synthesis. Cells were treated with 50 μM compounds for 24 hr. During the last hour, cells were pulse-labeled with EdU, stained with the Click-iT EdU Alexa Fluor 488 Assay Kit, counterstained with 7-AAD, and subsequently analyzed by flow cytometry. EdU-positive cells were gated to determine the percentage of the S phase cell population. The bivariate plots of Alexa Fluor 488 (EdU-positive) and PE-Cy5 (7-AAD-positive) are shown.

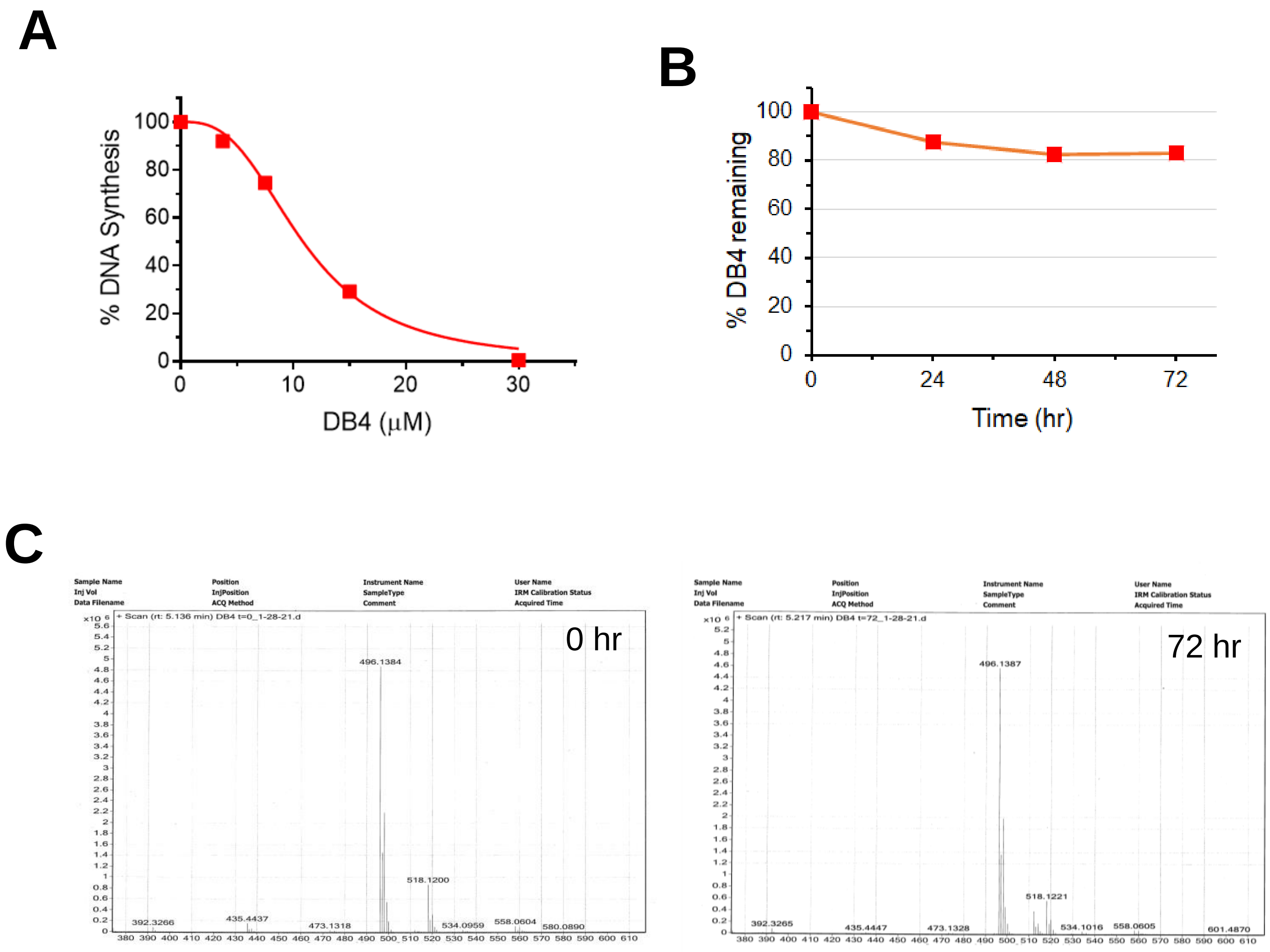

Fig S2. Chemical stability of DB4. DB4 remains mostly active and intact after 72 hr in the culture medium. The dose response curve of DB4 in DNA synthesis inhibition in PEO4 cells was generated as determined by EdU flow cytometric analysis (A). DB4 was added to the cell culture medium at 30 μM and incubated for 0, 24, 48, or 72 hr at 37°C. PEO4 cells were treated with these media for 24 hr. The level of DNA synthesis inhibition was determined to interpolate and calculate %DB4 remaining compared with the control at 0 hr (B). DB4 (MW=496.1) was added to the culture medium at 50 μM for 0, 24, 48, and 72 hr at 37°C. Mass spectrometry was run to detect the level of the 496.13 ion. The data at 0 and 72 hr are shown (C).

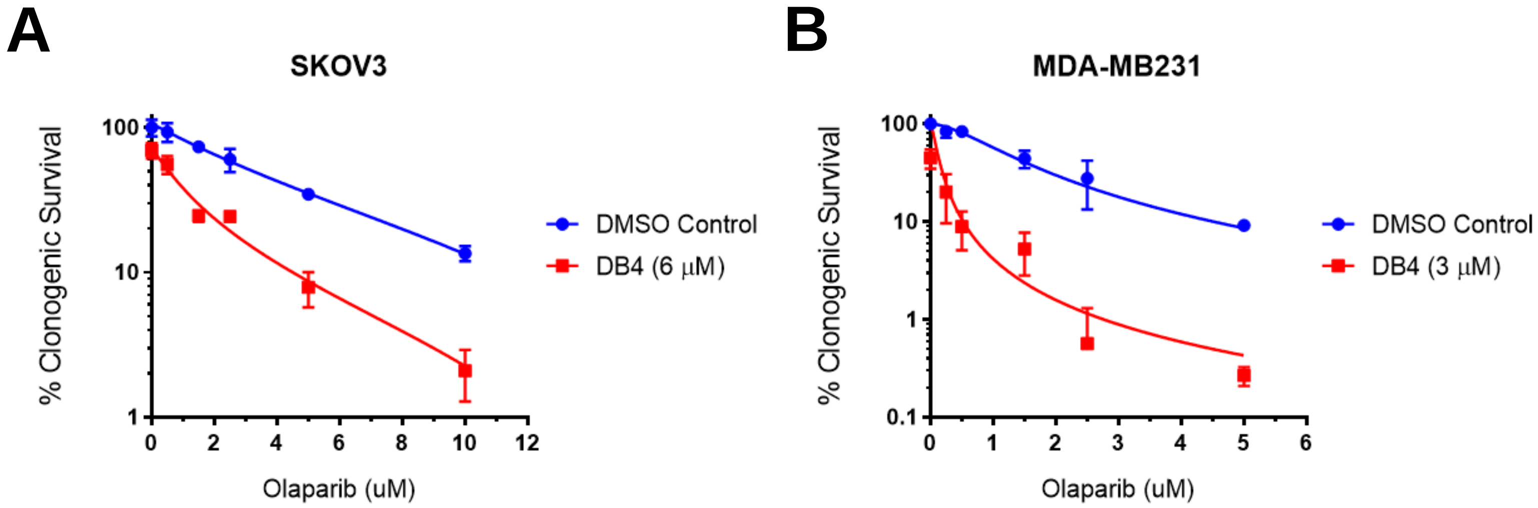

Fig S3. DB4 sensitizes BRCA-wild type SKOV3 (A) and MDA-MB231 (B) cells to olaparib. Cells were plated for 24 hr, pre-treated with DB4 for 1 hr, and then treated with various concentrations of olaparib for 14 days. Colonies were stained and determined for clonogenic survival.

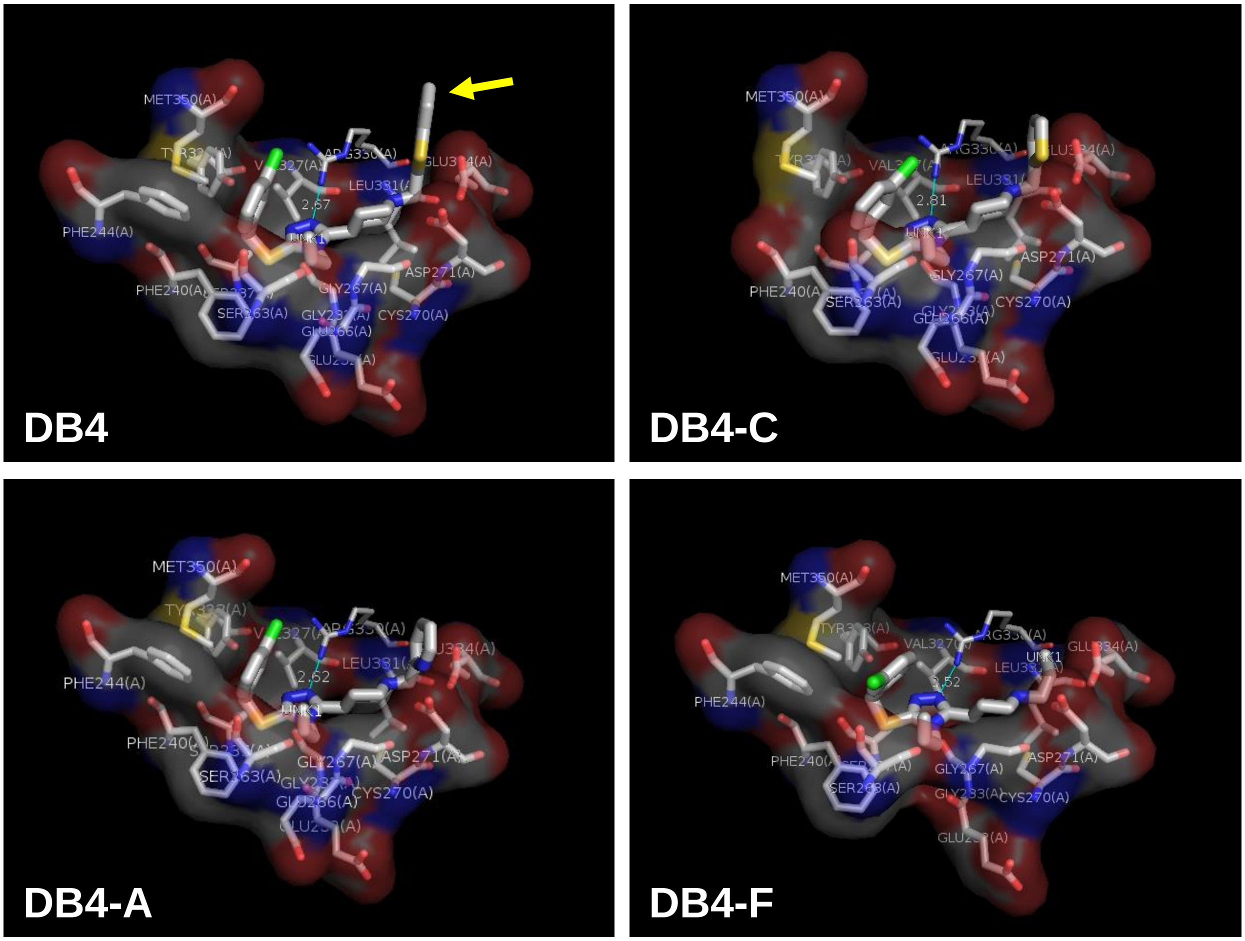

Fig S4. 3D representations of DB4 and DB4 analog docking poses in the triapine binding pocket. Yellow arrow indicates the protrusion of the benzene ring of the benzothiazole group of DB4 above the binding pocket.

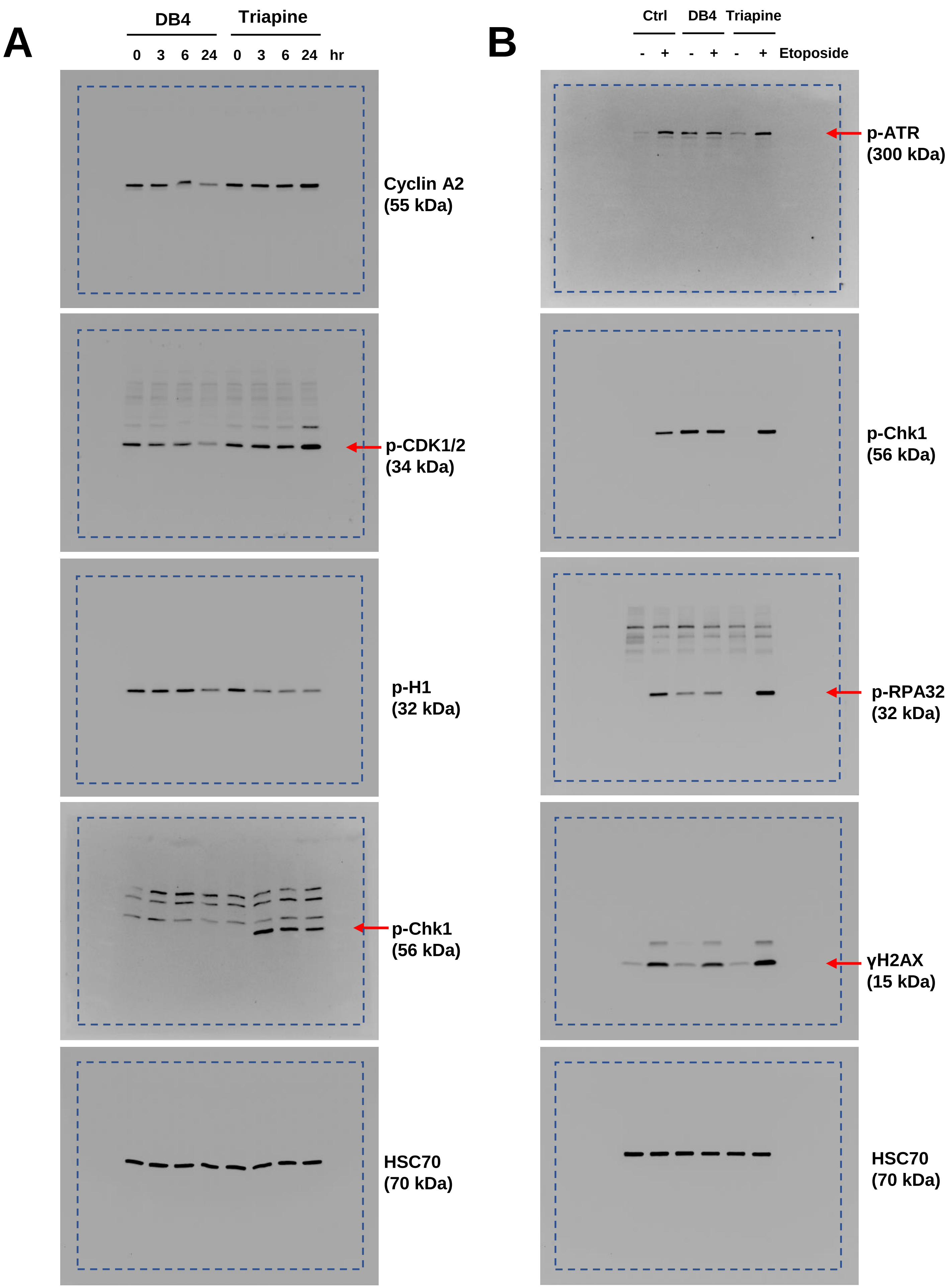

Fig S5. Full length gel images of Fig 4C (A) and 4E (B). Dash lines indicate the border of blots

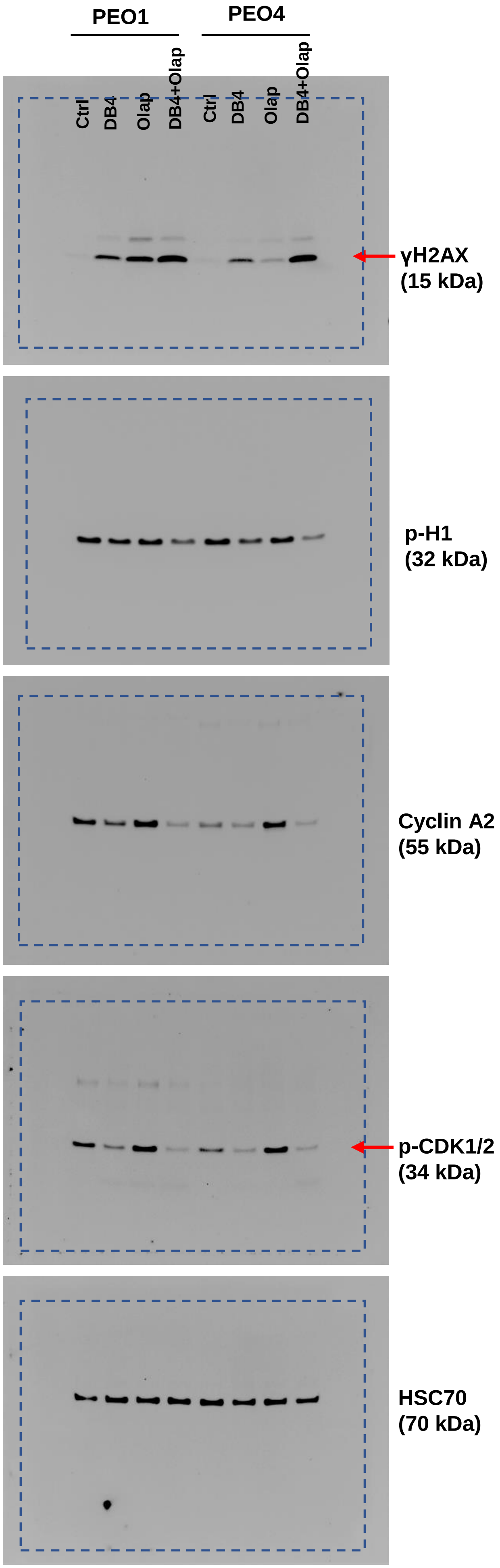

Fig S6. Full length gel images of Fig 5E. Dash lines indicate the border of blots

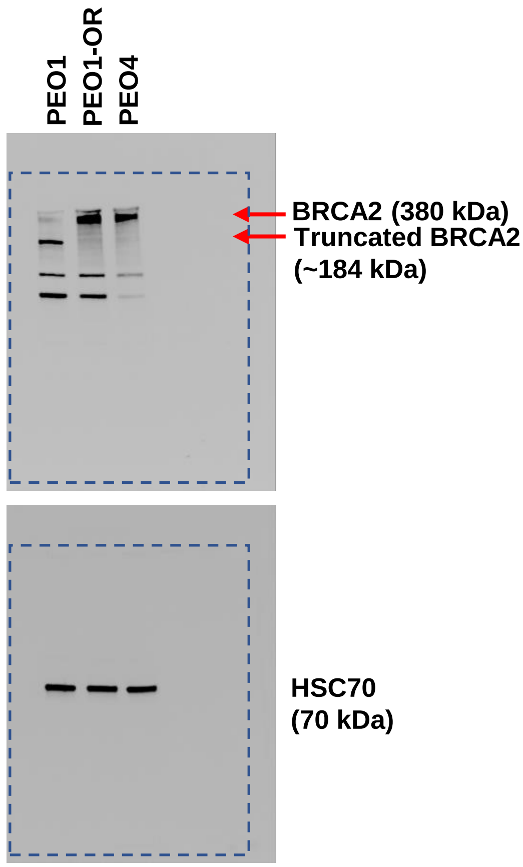

Fig S7. Full length gel images of Fig 6E. Dash lines indicate the border of blots
